# Host identity drives the assembly of phytoplankton microbiomes across a continental-scale environmental gradient

**DOI:** 10.1093/ismejo/wraf083

**Published:** 2025-04-30

**Authors:** Patricia Signe White, Taryn Y Broe, Mirte C M Kuijpers, Jonathan R Dickey, Sara L Jackrel

**Affiliations:** Department of Ecology, Behavior, and Evolution, University of California San Diego, La Jolla, CA 92093-0116, United States; Department of Integrative Biology, University of California Berkeley, Berkeley, CA 94720-2284, United States; Department of Ecology, Behavior, and Evolution, University of California San Diego, La Jolla, CA 92093-0116, United States; Department of Ecology, Behavior, and Evolution, University of California San Diego, La Jolla, CA 92093-0116, United States; Department of Ecology, Behavior, and Evolution, University of California San Diego, La Jolla, CA 92093-0116, United States; Department of Ecology, Behavior, and Evolution, University of California San Diego, La Jolla, CA 92093-0116, United States

**Keywords:** host–microbiome, microbiome assembly, phytoplankton, thermal ecology, host fitness

## Abstract

Host-associated microbiomes often promote host health, yet the key drivers of microbiome assembly and its consequences for host fitness remain unclear. We aimed to determine the relative roles of host identity versus the environment in driving host–microbiome assembly and the consequences of this variation in assembly for host fitness, which may help predict the resilience of host-associated microbiomes and host health amidst fluctuating environmental conditions. Here, we tracked microbiome assembly in association with initially axenic phytoplankton when incubated in seawater originating from four nearshore locations along a continental-scale environmental gradient of North America. Microbiome assembly was highly deterministic. Unexpectedly, host species identity was the overwhelming driver of microbiome community assembly despite continental-scale variation in the environment. Although secondary to host identity, the environment was a significant driver of microbiome assembly for each species evaluated, which, in turn, conferred cascading effects on host fitness as shown by thermal tolerance growth assays. We also found that host-specific microbiomes had host-specific fitness effects, particularly under thermally stressful conditions. Overall, our results advance our understanding of microbiome assembly by empirically demonstrating that although variation among host microbiomes imparted by the local environment has significant implications for host health, the host species is the overwhelming driver of microbiome assembly regardless of wide-scale variation in the environment.

## Introduction

All eukaryotes harbour microbiomes, and there is increasing evidence that microbiomes can drive host physiology, fitness, behaviour, and ecology [1–6]. Determining what regulates host microbiome composition is essential for understanding how hosts maintain health and resist disease amid environmental stressors [1, 7–11]. Further, host microbiomes hold broad applications, including their potential to mitigate climate change, enhance ecosystem management, support wildlife conservation, and improve human health.

Host identity and the surrounding environment are two of the most significant regulators of host microbiome composition [12–23]. Host identity, including phenotypic traits encoded by the host genome, influences microbiome composition by filtering which taxa can persist. The physiochemical properties of the host habitat, such as pH, further promote the recruitment of bacterial taxa that confer survival advantages under those conditions [24]. In contrast, the surrounding environment shapes the microbiome by providing both the source community of microbes available to assemble with a host, and abiotic factors, such as nutrients and temperature, that sustain them.

Most studies comparing the roles of host identity and the environment in shaping microbiome composition rely on observational data. Although such studies have limitations, research across taxa—plants, invertebrates, and vertebrates, including humans—has demonstrated that host identity, especially genetics, plays a key role in microbiome composition [12–19, 21, 22]. However, among the few studies explicitly comparing host and environmental effects, some suggest that environmental factors exert a stronger influence [16, 20, 23]. Therefore, empirical studies are needed to resolve the relative contribution of these two factors in shaping microbiome composition. Moreover, if variation among host microbiomes is largely the result of host identity and the environment, then it is important to understand the functional consequences of these two pools of variation on host fitness. For instance, species-specific microbiomes confer fitness benefits under abiotically stressful conditions in some insect hosts [8], whereas locally acquired microbiomes have enhanced host fitness relative to nonlocal microbiomes in certain invertebrates [8, 25]. Yet, the relative importance of these two factors in driving microbiome variation and host fitness remains unknown.

We test the relative importance of host identity versus the environment on microbiome assembly using single-celled eukaryotic phytoplankton as an experimentally tractable system, allowing us to completely remove (i.e., render axenic) and reassemble host microbiomes under controlled conditions. Phytoplankton are surrounded by a nutrient-rich extracellular diffusive boundary layer teeming with bacteria [26, 27]. Phytoplankton and their microbiomes engage in diverse interactions that can influence host health, including parasitism, competition for inorganic nutrients, and symbiotic exchanges of vitamins [28]. Due to the short generation times of phytoplankton, this model microbial system can be used to quantify how environmentally driven microbiome variation affects host fitness. Further, to test the fitness effects of host-specific microbiomes, we use reciprocal microbiome transplants between phytoplankton host species.

In this study, we expose six species of axenic marine phytoplankton to seawater collected from four sites spanning ~30° of latitude along the nearshore of western North America to test the relative contributions of host identity and the environment on microbiome assembly. In previous work using a similar experimental approach, we had demonstrated that microbiome assembly is deterministic and predominantly driven by host species identity rather than environmental factors [29]. However, that proof-of-concept study used water from three naturalized ponds within a small, 120 m^2^ artificial pond facility at a university field station. To more rigorously test the relative importance of host species identity versus the environment, we expanded the scope by sourcing seawater from a continental-scale gradient. Additionally, we assessed the fitness implications of both environmental and host-specific components of the microbiome. First, we tested whether microbial communities assembled from across this environmental gradient, which likely include bacterial taxa adapted to differing thermal conditions, influence the thermal tolerance of their phytoplankton hosts. This was achieved by measuring phytoplankton population growth across a range of temperatures, spanning those found across the environmental gradient, as well as higher temperatures approaching the thermal limits of most marine phytoplankton. Second, we evaluated whether host-specific microbiomes conferred greater host fitness advantages, particularly under thermal stress.

Understanding the drivers of microbiome assembly and their functional implications for their hosts provides insights into how host–microbiome interactions might respond to environmental changes, including altered geographic ranges and abiotic stressors caused by climate change. By elucidating these dynamics, we can better anticipate ecosystem responses to global changes, with applications in conservation, ecosystem management, and climate change mitigation.

## Materials and methods

### Species pool

Axenic cultures of six unicellular eukaryotic marine diatom species were obtained from the Bigelow National Center for Marine Algae and Microbiota (East Boothbay, ME, USA). Diatoms, which are a phylum of phytoplankton characterized by their silica cell walls, used in this study included: *Achnanthes brevipes*, *Nitzschia punctata*, *Navicula salinicola*, and *Phaeodactylum tricornutum*, all of which belong to the Bacillariophyceae, and *Ditylum brightwellii* and *Thalassiosira pseudonana,* from the Mediophyceae. We used strains from the western coast of North America when possible, and report thermal conditions and coordinates of each strain’s collection site in Table S1. The axenic status of all cultures was verified using fluorescence microscopy and attempted isolation of bacterial heterotrophs on marine broth agar.

### Microbiome assembly experiment

To investigate whether diatoms recruited host-specific microbiomes across an environmental gradient, we submerged high-density axenic monocultures into seawater using our previously established methods [29]. Diatom cultures were enclosed in jars sealed with bacteria-permeable membranes and submerged in aquarium tanks filled with seawater. This setup allowed bacteria from the surrounding seawater to migrate in response to diatom exudates, selectively entering the jars containing one of the six diatom species. We completed this study under uniform temperature and lighting conditions for all sites. Although site-specific incubation conditions (e.g., temperature, light intensity) might better preserve environmentally sensitive or site-specific bacterial taxa, they would also alter host physiology, confounding interpretations of whether observed differences in microbiome assembly were due to the environment versus the host response. We therefore chose standardized conditions to minimize physiological variation in hosts across sites. This decision allowed us to control for effects of host physiology—such as growth rate, cell senescence, and exudate production—which are known to regulate bacterial chemotaxis and microbiome assembly in phytoplankton [28, 30]. While this approach is not without limitations, it provided a clear test of the relative contributions of host identity and environmental origin to microbiome structure.

We sampled water at four locations along the western coast of North America: San Diego Bay (CA, USA), Bodega Bay Marine Lab (CA, USA), Friday Harbor Lab (WA, USA), and Kachemak Bay (AK, USA) (see Fig. S1 and Table S2 for site details). On 15 June 2021, 60 L of water was collected at each site, with 20 L from the surface, 20 L from the benthic region (see Table S2 for maximum depths), and 20 L from the mid-depth. To minimize microbial community shifts during transportation, water was shipped in Cubitainers within triple-layer, thermally insulated liners to University of California San Diego (UCSD), arriving by 8 a.m. on 16 June 2021. Upon arrival, water from the three depths at each site was mixed. Approximately 3.0 L from each site was filtered through in-line 3.0 and 0.22 μm filters, with filters stored at −80°C until DNA extractions. Seawater was also reserved for salinity and nutrient analysis (see Table S2). The remaining ~57 L of seawater per site was transferred to 10 G aquarium tanks equipped with oxygen bubblers and maintained under 16:8 h light–dark fluorescent lighting at 20°C. We prepared axenic diatom cultures grown in L1 phytoplankton growth medium for microbiome assembly. Diatoms were inoculated into 100 ml of L1 at 100 000 cells/ml in 4 oz glass jars sealed with mixed cellulose ester filters having a pore size of 3.0 μm and a diameter of 90 mm. These filters retained diatom cells but permitted passage of smaller bacterial cells into the jar. Due to the lower density of stock culture, *D. brightwellii* was inoculated at 4000 cells/ml. Jars were affixed to the walls of aquariums using ceramic magnets and epoxy to prevent debris from accumulating on the 3.0 μm filters, which could obstruct seawater and bacterial passage. Five replicates per species per location were deployed, resulting in a total of 120 jars (6 species × 5 replicates × 4 locations). The spatial arrangement of jars within aquariums was randomized, and jars were submerged at least 5 cm below the water surface for 82 h to facilitate microbiome assembly (Fig. S1).

At the end of the incubation period on 20 June 2021, we preserved aliquots from each culture to characterize the diatom-associated bacterial communities using 16S rRNA sequencing. To isolate the particle-associated (i.e. diatom-associated) microbiome, 75 ml of each culture was filtered through 3.0 μm filters, and the filters were stored at −80°C until DNA extraction. The remaining culture volumes were transferred to flasks containing L1 media and incubated under 16:8-h Sun White LED lighting (Active Grow) at 20°C and 80 rpm. These flasks were maintained as stock cultures for subsequent growth measurements.

### Seawater incubation experiment

To assess how the incubation conditions influenced bacterial community composition over time, we evaluated seawater collected from the four geographic locations used in the microbiome assembly experiment. Specifically, we sought to confirm that bacterial communities from each geographic location remained distinct throughout the 82-h incubation period, despite identical temperature and lighting conditions. Additionally, we aimed to determine whether diatom-associated microbiomes that assembled after 82 h were compositionally distinct from seawater bacterial communities sampled across this temporal gradient. To achieve this, we conducted a second seawater incubation experiment on 23 April 2024. Following the previously described methods, seawater samples were shipped to UCSD, maintained in two aquariums per location under uniform conditions, and analysed for bacterial community composition and nutrient concentrations. Sampling via in-line 3.0 and 0.22 μm filtration for 16S rRNA amplicon sequencing and nutrient analysis was performed at 0, 24, 48, 72, and 82 h. We collected one replicate for each measurement per aquarium per time point, with the exception of salinity, which was measured in triplicate.

### Thermal performance fitness assays

We evaluated whether diatom-associated microbiomes recruited from seawater of different geographic origins conferred varying levels of thermal tolerance. We measured diatom population growth of each culture at five temperature conditions spanning a gradient of 24°C, including 6°C, 12°C, 18°C, 24°C, and 30°C. These temperatures were selected to reflect the mean environmental conditions across the collection sites, ranging from 6.6 ± 2.7°C in Kachemak Bay to 18.6 ± 2.6°C in San Diego Bay (Table S2). Temperatures up to 30°C were also included to approach the critical thermal maximum for marine diatoms, which typically falls between 30°C and 35°C, and to encompass the maximum surface temperature recorded at our collection sites (24.8°C in San Diego Bay, 2021) [31, 32]. This design allowed us to test whether the microbiome’s origin significantly influences host fitness under high abiotic stress, as observed in other systems [8]. Population growth was monitored over the full growth trajectory, from the lag phase to carrying capacity, which for the marine diatoms used in this study, tends to occur by 2–3 weeks postinoculation, depending on environmental factors such as incubation temperature and nutrient composition of the growth media.

We selected three diatom species from our microbiome assembly experiment that exhibited the most robust growth patterns in a 96 deep-well plate format: *P. tricornutum*, *N. punctata*, and *N. salinicola*. This robust growth may have been due to geographic origin, as these three taxa originated from the lowest latitudes among our species pool (see Table S1). Cultures of these diatoms, each with microbiomes assembled from the four seawater origins (12 cultures), were inoculated into 1.8 ml of L1 at a density of 5000 cells/mL. Growth assays were conducted in deep-well culture plates nested within pans placed onto Torrey Pines Scientific Ecotherm digital chilling/heating dry baths, which maintained precise temperature conditions. To minimize desiccation, we maintained high humidity over the duration of these assays by anchoring deep-well plates to the bottom of aluminium bread pans filled with water. Each culture was tested at five temperatures with three technical replicates per condition, resulting in 60 combinations arranged in a randomized design. Due to the inability to obtain exponential growth curves for most cultures at 6°C, this temperature was excluded from further analysis. Assays were conducted from 3 July to 2 August 2021, which was within 2 weeks of the initial microbiome assembly experiment. Growth was tracked using chlorophyll-a fluorescence (435/680 nm excitation emission) as a proxy for cell density using a Tecan Infinite F Nano+ plate reader and measured every 2–3 days from Day 0 to Day 24 and again on Day 29. By this time, most cultures had reached carrying capacity, as indicated by asymptotic growth curves.

### Microbiome transplant assays

To evaluate whether species-specific microbiomes confer host-specific fitness benefits, we repeated a subset of the thermal performance assays after conducting reciprocal microbiome transplants. Specifically, we aimed to create a thermal mismatch by testing diatoms with microbiomes originating from Friday Harbor, WA, and Kachemak Bay, AK, under the two warmest temperature conditions: 24°C and 30°C. By using the diatoms *N. salinicola* and *P. tricornutum*, which originated from the lowest latitude in our species pool, the 24°C condition is within the upper thermal range that these strains may have experienced at their geographic origins, whereas 30°C exceeds the maximum recorded temperatures (Table S1). This approach was motivated by prior research in insects demonstrating that fitness benefits conferred by maternally provisioned microbiomes became more apparent under abiotic stress [8]. Similarly, our earlier work with freshwater phytoplankton found no evidence of species-specific fitness effects of algal microbiomes under benign growth conditions, prompting us to revisit this question in the context of thermal stress [29].

We conducted reciprocal microbiome transplants between cultures of *P. tricornutum* and *N. salinicola*, each with microbiomes initially assembled from Friday Harbor. A second set of transplants was performed between these diatom species with microbiomes assembled from Kachemak Bay. To obtain the microbiome communities for transplantation, we followed our previously established methods [29]. Briefly, 10 ml aliquots of cultures, standardized to 5000 diatom cells/ml, were sonicated on ice to dislodge microbes from the diatom mucilage. Sonication was performed in three 30-s pulses at 20% amplitude with 1-min intervals between pulses using a Fisherbrand Model 50 Sonic Dismembrator. Samples were then centrifuged at 900 × g for 5 min, and the resulting supernatant containing the microbial cells was passed through a 3.0 μm filter to remove any residual diatom cells. Axenic diatoms were inoculated at 5000 cells/ml into 1.8 ml of media in 96-well deep well plates, with 10 μl of the bacterial community added to each well. To investigate the potential benefits of bacterial communities under high thermal stress, we completed six replicates of each combination in standard L1 media and six replicates in L1 media lacking vitamin B12, as phytoplankton-associated bacteria are hypothesized to enhance host fitness at high temperatures by supplying this vitamin [33]. Wells were arranged in a randomized design across plates and incubated at 24°C or 30°C. Fluorescence measurements were taken every 2–3 days for 24 days, at which point most cultures reached carrying capacity as indicated by asymptotic growth curves. These assays were shorter than the initial thermal performance assays, as populations generally reached carrying capacity more rapidly at these elevated temperatures.

### Amplicon sequencing, analysis, and statistics

We analysed bacterial community composition via amplification and high-throughput sequencing of the V4 region of the 16S rRNA genes. We describe all details of DNA extraction, sequencing, and analysis via the QIIME2-data2 pipeline in our supplementary methods. We compared bacterial taxonomic richness and Shannon diversity across diatom species and seawater origins. Bacterial community composition was assessed using Principal Coordinates Analaysis (PCoA) ordinations and permutational multivariate analysis of variance (PERMANOVA) on Bray–Curtis distance matrices, with Species and Seawater Origin as fixed effects. To minimize arching artefacts caused by the Guttman effect in ordinations, we applied the step-across flexible shortest path correction in the vegan package [34]. Pairwise differences among host species and seawater origins were tested using Tukey’s *post hoc* tests. All statistical analyses and visualizations were generated using the vegan, phyloseq, and ggplot2 packages in R [35, 36].

Population growth over time was quantified using the area under the curve (AUC) derived from logistic equation fits to the full-length chlorophyll-a fluorescence curves, implemented via the growthcurver package [37]. AUC describes the full growth trajectory from the exponential phase through maximum density. The exponential rate of growth (μ) is a valuable indicator of evolutionary fitness, revealing per-capita growth rate in the absence of intraspecific competition. However, bacteria often promote host growth via nutrient recycling [28]; consequently, their effects might be strongest during late-phase growth once intraspecific competition increases, thereby affecting maximum density. AUC encompasses both of these components: the best-fitting model of AUC with the lowest AIC score was the combination of maximum density, μ, and their interaction (marginal *R*^2^ = 0.80), exceeding the predictive power of models containing only a single growth parameter or no interaction term. We also report both parameters independently. The exponential growth rate was estimated from the logistic model fits, but to improve accuracy, curves were trimmed to exclude data after cultures reached carrying capacity, which occurred between 12 and 29 days, depending on conditions. Although contributing significantly to our best fitting model, μ alone was a weak predictor of AUC (*R*^2^ = 0.38, *P* = 0.089). Maximum density, or carrying capacity, was represented by the highest recorded relative fluorescence unit (RFU) value over the full-length growth curve. Maximum density alone strongly predicted AUC (*R*^2^ = 0.77, *P* < .001). We used two-way analyses of variance (ANOVAs) to determine the independent and interactive effects of Temperature and Seawater Origin on each of the three dependent metrics (AUC, μ and maximum density) for our thermal performance assays, and the effects of Species-Specific Microbiome, Algal Host Species, and Seawater Origin for our microbiome transplant assays. To meet the assumptions of homogeneity of variance for ANOVA models, square root or Box–Cox transformations were applied to dependent variables when necessary. To identify parsimonious ANOVA model fits, structures were simplified when possible by dropping nonsignificant interaction terms [38, 39].

## Results

**Figure 1:**
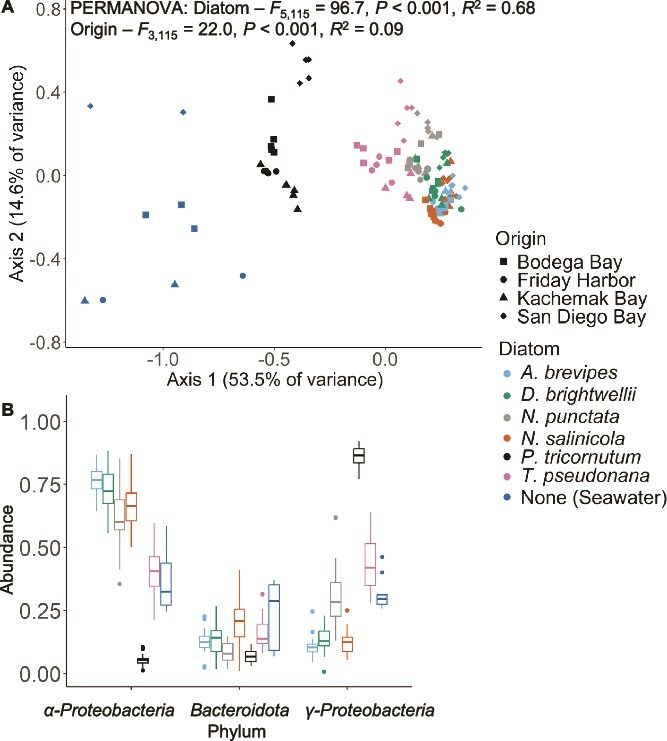
(A) Bacterial community composition described using a Bray–Curtis distance metric exhibited specificity to the diatom–host species immediately following an 82-h incubation period of initially axenic marine diatoms in seawater. Seawater was collected from across a continental-scale thermal gradient spanning San Diego Bay, CA, in the South (32.7°N) to Kachemak Bay, AK, in the North (59.6°N). Bacterial composition differed significantly between all diatom species via pairwise *post hoc* tests where *P* < .001. (B) Mean proportional abundances of common phyla associated with each of the six species of diatoms and found in seawater (see Fig. S3 for rare phyla). Host specificity could be explained in part by the dominance of γ*-Proteobacteria* in the cultures of *P. tricornutum* versus the dominance of *α-Proteobacteria* in the other five species of diatoms. Seawater samples are included in the ordination but not the statistics to focus on separation among species of diatom.

The assembly of microbiome communities in association with six initially axenic marine diatoms was driven by both host species identity and the environment. Of these two factors, host species identity emerged as the dominant driver of microbiome community composition (Fig. 1A; PERMANOVA: Diatom Species *F*_5,115_ = 96.7, *P* < .001, *R*^2^ = 0.68, all *post hoc* pairwise comparisons, *P* < .001). In contrast, the environment, represented by microbial source communities from a continental-scale gradient ranging from San Diego Bay, CA, in the south to Kachemak Bay, AK, in the north, had a significant but smaller effect on microbiome assembly (Fig. 1A; PERMANOVA: Seawater Origin *F*_3,115_ = 22.0, *P* < .001, *R*^2^ = 0.09; see Fig. S2 for results at higher rarefaction depth).

A component of this host−species-specific microbiome assembly was the relative abundances of *γ-Proteobacteria* versus *α-Proteobacteria*. For example, *P. tricornutum* microbiomes were dominated by *γ-Proteobacteria*, whereas the other five species had a greater proportion of *α-Proteobacteria* (Fig. 1B). Other phyla also varied across host species; for example, we observed a higher prevalence of *Planctomycetota* and *Campylobacterota* in *P. tricornutum* microbiomes (Fig. S3). Microbiome assembly also varied among taxa known to regulate biogeochemical cycles, for instance, *Sulfitobacter* sp. comprised 1.4% ± 0.33 standard error (SE) of the *P. tricornutum* microbiome but <0.05% in the microbiomes of other diatom species (Table S3). Microbiomes of host species also differed in alpha diversity, with *P. tricornutum* microbiomes harbouring the greatest taxon richness and *T. pseudonana* microbiomes having the greatest Shannon diversity (Fig. S4).

These species-specific diatom microbiomes that assembled after 82 h were also distinct from the surrounding seawater communities. Diatom microbiomes differed from the initial bacterial communities present in seawater at *T*_0_ (Fig. 1; all pairwise *post hoc* comparisons, *P* values < .001). Moreover, these microbiomes were distinct from all seawater bacterial communities sampled over the 82-h temporal gradient, during which seawater from four geographic locations was incubated under identical temperature and lighting conditions (Fig. S5; all pairwise *post hoc* comparisons, *P*-values < .001). Throughout the 82-h seawater incubation, bacterial community composition was primarily driven by seawater origin, with timepoint serving as a secondary driver (Fig. S6). Specifically, bacterial community composition differed between all seawater origins, independent of timepoint (pairwise *post hoc* comparisons, *P*-values < .001). In contrast, bacterial community composition shifted between *T*_0_ and subsequent timepoints (*P-*values < .01) but then stabilized from 24 h through the end of the 82-h incubation experiment (*P-*values > .05). Over this time, nutrient concentrations, including NO_3_, NO_2_, NH_4_, and PO_4_, generally declined but exhibited variability across geographic locations (Fig. S7).

**Figure 2:**
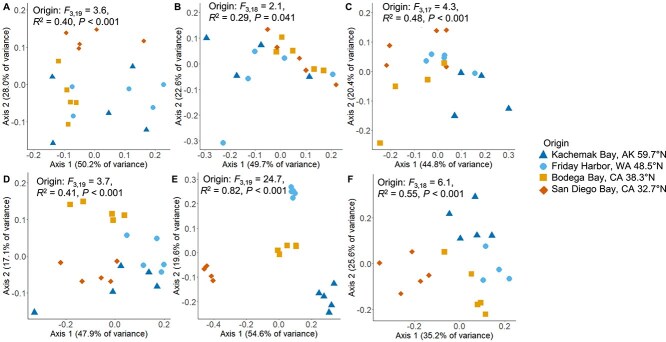
Bacterial community composition described using a Bray–Curtis distance metric shows host species identity as the dominant driver of the diatom microbiome. However, within each of the six species of diatom, seawater origin was a strong predictor of microbial community composition. (A) *A. brevipes*, (B) *D. brightwellii*, (C) *N. punctata* (D) *N. salinicola*, (E) *P. tricornutum*, and (F) *T. pseudonana*. Seawater was collected from across a continental-scale gradient spanning San Diego Bay, CA, in the South to Kachemak Bay, AK, in the North.

Although host species identity was the primary determinant of microbiome assembly across our multispecies dataset, the environment significantly influenced microbiome composition within each of the six marine diatom species (Fig. 2). Specifically, microbiomes assembled by all six species varied significantly depending on seawater origin (Fig. 2; PERMANOVA with Seawater Origin as the treatment effect: *A. brevipes F*_3,19_ = 3.6, *R*^2^ = 0.40, *P* < .001; *D. brightwellii F*_3,18_ = 2.1, *R*^2^ = 0.29, *P* = .041; *N. punctata F*_3,17_ = 4.3, *R*^2^ = 0.48, *P* < .001; *N. salinicola F*  _3,19_ = 3.7, *R*^2^ = 0.41, *P* < .001; *P. tricornutum F*_3,19_ = 24.7, *R*^2^ = 0.82, *P* < .001; *T. pseudonana F*_3,18_ = 6.2, *R*^2^ = 0.55, *P* < .001). Within *P. tricornutum* cultures, which were characterized by a predominance of *γ-Proteobacteria*, the seawater origin was also a significant predictor of microbiome composition when considering only the *γ-Proteobacteria* (Fig. S8A; PERMANOVA *F*_3,19_ = 27.4, *R*^2^ = 0.84, *P* < .001). Although a strain of *Vibrio* sp*.* was the most abundant in the *P. tricornutum* microbiome (μ = 27.8% ± 3.8 SE), there were 19 distinct strains of *γ-Proteobacteria* regularly found in association with this host (Fig. S8B and C, Table S3). Furthermore, diatom microbiomes assembled in seawater from Kachemak Bay exhibited the lowest taxon richness and Shannon diversity across all seawater origins (Fig. S4).

**Figure 3:**
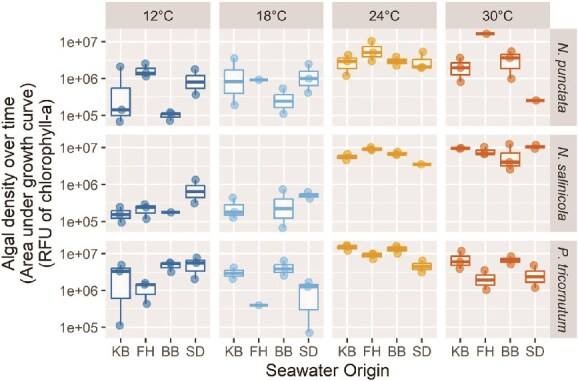
The origin of the seawater used for diatom microbiome assembly and incubation temperature often had significant growth effects for the diatom host. Three species of diatoms with microbiomes assembled from one of four seawater collection sites (KB = Kachemak Bay, AK; FH = Friday Harbor, WA; BB = Bodega Bay, CA; SD = San Diego Bay, CA) were inoculated into L1 media and incubated at one of four temperatures for 29 days. Diatom density over time, as estimated via chlorophyll-a fluorescence, is reported as the total area under the fitted logistic growth curve. Two-way analysis of variance tests evaluated temperature and seawater origin as fixed effects for each species. *N. punctata:* Temperature—*F*_3,22_ = 8.8, *P* < .01, Origin—*F*_3,22_ = 7.9, *P* < .01, Temperature × Origin Interaction—*P* < .01. *Navicula salinicola:* Temperature—*F*_3,20_ = 42.6, *P* < .01, Origin—*F*_3,01_ = 0.8, *P* = .50, Temperature × Origin Interaction—*P* = .19. *P. tricornutum:* Temperature—*F*_3,25_ = 28.3, *P* < .01, Origin—*F*_3,25_ = 8.34, *P* < .01, Temperature × Origin Interaction—*P* = .053. No data are shown for cultures of *N. salinicola* with a microbiome from Friday Harbor incubated at 18°C due to all replicates failing to attain exponential growth.

The four seawater collection sites differed substantially in environmental conditions, including annual mean temperature (18.6°C ± 2.6 SD in San Diego Bay versus 6.6°C ± 2.7 SD in Kachemak Bay) and nutrient concentrations (see Table S2). We found that the corresponding microbiomes assembled from these sites for each diatom species often conferred measurable differences in host fitness, as determined by the AUC of fitted logistic growth curve models (Fig. 3: ANOVA—Seawater Origin—*N. punctata*: *F*_3,22_ = 7.9, *P* < .01; *N. salinicola*: *F*_3,20_ = 0.8, *P* = .50; *P. tricornutum*: *F*_3,25_ = 8.3, *P* < .01). Fitness also differed markedly across temperatures for all three diatom species (Fig. 3: Temperature—*N. punctata*: *F*_3,22_ = 8.8, *P* < .01; *N. salinicola*: *F*_3,20_ = 42.6, *P* < .01; *P. tricornutum*: *F*_3,25_ = 28.3, *P* < .01). Further, two of the three diatom species showed a temperature × seawater origin interaction (*N. punctata*: *P* < .01, *N. salinicola*: *P* = .19, *P. tricornutum*: *P* = .053). However, rather than our expectation that the thermal origin of seawater used to assemble a host microbiome would confer greatest host fitness at similar temperatures, we often observed the opposite trend: microbiomes assembled from Kachemak Bay seawater tended to confer the lowest host fitness at the coldest temperature, 12°C, whereas microbiomes assembled from San Diego Bay seawater tended to confer the lowest host fitness at the warmest temperature tested, 30°C. Additionally, considering that *N. punctata* originated from San Diego, the strong temperature by seawater origin interaction for this species may indicate a home-site advantage. *Nitzschia punctata* reached higher AUCs when grown with its local San Diego microbiome at San Diego–relevant temperatures of 18°C and 24°C compared to other diatoms grown under these conditions (Table S4). In addition to AUC trends, we found that seawater origin and temperature often influenced host maximum density (Fig. S9, Seawater Origin: *N. punctata*: *F*_3,32_ = 5.0, *P* < .01; *N. salinicola*: *F*_3,31_ = 0.6, *P* = .64; *P. tricornutum*: *F*_3,34_ = 6.6, *P* < .01; Temperature: *N. punctata*: *F*_3,32_ = 12.2, *P* < .01; *N. salinicola*: *F*_3,31_ = 39.8, *P* < .01; *P. tricornutum*: *F*_3,34_ = 11.2, *P* < .01). We also found that seawater origin and temperature influenced the exponential growth rate of *P. tricornutum* (Fig. S10: Seawater Origin *F*_3,25_ = 2.8, *P* = .061; Temperature *F*_3,25_ = 9.3, *P* < .01; Origin × Temperature Interaction *P* < .01). For full growth curve data for all populations, see Fig. S11.

**Figure 4:**
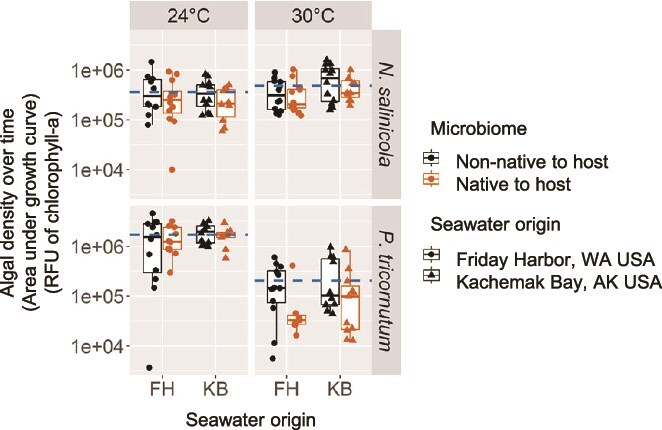
Host-specific microbiomes conferred host-specific fitness effects under thermal stress, specifically during the first half of growth. Our proxy for host fitness for each of the two species of diatoms is population density, as estimated via chlorophyll-a fluorescence measured over time at one of two temperatures, and is reported as the total area under fitted logistic growth curves. Results from the first 12 days of the 24-day growth curve are shown to highlight the stronger effect identified during early-stage growth (see Fig. S12 for results on full growth curve). Axenic diatoms were inoculated with either their own native microbiome or the microbiome of the other species (i.e. non-native). The analysis of variance test evaluated Species-specific Microbiome (i.e. native or non-native to the host), Algal Host Species (i.e. *P. tricornutum* or *N. salinicola*), seawater origin (Friday Harbor, WA vs. Kachemak Bay, AK), and Temperature (24 vs. 30°C) as fixed effects with media type as an error term (i.e. with or without extra vitamin B12, see Fig. S13 for separation of replicates grown with and without vitamin B12). Species-specific Microbiome: *F*_1,169_ = 5.5, *P* = .021; Seawater Origin: *F*_1,169_ = 3.6, *P* = .059; Temperature: *F*_1,169_ = 73.9, *P* < .001; Algal Host Species: *F*_1,169_ = 6.8, *P* = .010; Temperature × Algal Host Species Interaction: *P* < .01. Tukey’s one-way *post hoc* comparison for Species-specific Microbiome: *t*_169_ = 2.36, *P* < .01. Dashed lines indicate mean diatom density for each facet.

To evaluate the fitness effects conferred by a diatom’s species-specific microbiome compared to the microbiome of a different diatom species, we conducted reciprocal microbiome transplants between *N. salinicola* and *P. tricornutum*. These two host species exhibited significant differences in their microbiomes, with the *N. salinicola* microbiome predominantly composed of α-Proteobacteria and the *P. tricornutum* microbiome dominated by γ-Proteobacteria (Fig. 1B). We refer to this species specificity of the microbiome when using the term “native” microbiome. However, as both diatoms originate from low latitudes, the species-specific microbiomes assembled from high-latitude sources likely differ from those assembled within the original low-latitude ranges of these hosts. We found that fitness effects conferred by the native microbiome, measured as the AUC, were most evident during early-stage growth for both species (Fig. 4; Species-specific Microbiome: *F*_1,169_ = 5.5, *P* = .021; Seawater Origin: *F*_1,169_ = 3.6, *P* = .059; Temperature: *F*_1,169_ = 73.9, *P* < .001; Algal Host Species: *F*_1,169_ = 6.8, *P* = .010; Temperature × Algal Host Species Interaction: *P* < .01; see Fig. S12 for results across the full growth curve). Contrary to our expectations, non-native microbiomes often conferred greater fitness benefits, particularly under stressful temperature conditions. Specifically, *N. salinicola* exhibited poorer growth at 24°C compared to 30°C, especially with its native microbiome. Similarly, *P. tricornutum* grew more poorly at 30°C, particularly with its native microbiome. Comparable trends were observed in replicates grown in L1 media with or without added vitamin B12 (Fig. S13). Although we found no effects of the native versus non-native microbiome on the maximum density achieved by diatom hosts (Fig. S14; *P. tricornutum*: *P* = .56; *N. salinicola*: *P* = .71), the native versus non-native microbiomes did influence the exponential growth rate, with context-dependent effects based on temperature and host species (Fig. S15; *P. tricornutum*: Species-Specific Microbiome *F*_1,80_ = 5.1, *P* = .026; Species-Specific Microbiome × Temperature Interaction: *F*_1,80_ = 4.9, *P* = .030; *N. salinicola*: Species-Specific Microbiome *F*_1,87_ = 2.1, *P* = .15). Full growth trajectories are shown in Fig. S16. We also compare growth dynamics between the thermal performance and microbiome transplant assays in Fig. S17 and expand on how experimental design may have contributed to differences in growth rates between the two studies.

## Discussion

### Microbiome assembly and its effects on thermal performance

Although other studies have found host identity to be a significant driver of microbiome community composition [12, 14, 15], our results emphasize just how profoundly strong these effects of host identity are compared to the effects of the surrounding environment. Despite acquiring microbial source communities drawn from seawater spanning nearly 30° of latitude with substantial variation in nutrient content and *in situ* temperature, we observed that the effects of host identity far outweighed those of the surrounding environment. Although the environment played a significant role, it was clearly secondary to host identity in shaping microbiome composition. This pattern persisted despite marked differences in bacterial community composition across the four seawater sources, which remained distinct throughout our 82-h *ex situ* study. A caveat to this result is that the assembly experiment took place under uniform temperature and light conditions for all four seawater sources. Although seawater bacterial communities rapidly stabilized within ~24 h, this early shift could have been driven by a die-off among the most environmentally sensitive, site-specific bacteria. Future work needs to investigate whether the effects of environment on host microbiome assembly increases with the use of *in situ* or site-specific incubation conditions. If maintenance of temperature or other site-specific parameters does shift microbiome assembly, considering that these more sensitive taxa would be the first to experience population declines from climate change, future work would need to assess the implications of these assembly differences for host fitness. However, while employing site-specific incubation conditions could better maintain site-specific bacterial taxa, these conditions would also undoubtedly alter host physiology with cascading implications for microbiome assembly, making it essential to disentangle the effects of host physiology versus the surrounding environment on microbiome assembly.

Our findings highlight that phytoplankton microbiomes are not only distinct from free-living bacterial communities in the surrounding seawater, but also that microbiome assembly is a highly deterministic process, even across continental-scale variation in the environment. Recent studies have shown that exposure of axenic diatoms to bacteria can induce extensive transcriptional and metabolic changes in the host [40]. These physiological responses can include the production of secondary metabolites that foster the growth and attachment of symbiotic bacteria and also inhibit the colonization of nonsymbiotic bacteria [40]. Combined with our results that solidify the role of host identity as a key driver of microbiome assembly, we might therefore expect convergent physiological responses by a host species to their associated microbes across large-scale environmental gradients.

Strong host specificity of microbiome assembly has ecological and biogeochemical implications. For example, shifting distributions among host species would likely shift available niche space for host-specific bacteria, many of which may play roles in biogeochemical functions, such as the role of *Sulfitobacter* spp. in the sulphur cycle [41]. Therefore, even if environmental changes have only weak direct effects on bacterial community composition, indirect effects mediated through shifts in host distributions across environmental gradients could have cascading implications for ecosystem processes [42]. Climate change, which is expected to alter the relative abundance of diatoms versus smaller phytoplankton taxa, as well as the proportion of different species within diatoms, could further amplify these cascading effects [43, 44]. Therefore, these findings underscore the need to integrate host-specific microbiome dynamics into models of bacterial-mediated ecosystem functions and biogeochemical cycling under climate change.

Although the environment played a secondary role in shaping microbiome composition, it had clear effects on host fitness. Seawater origin influenced thermal performance in two of the three diatom species tested, particularly in terms of AUC and maximum density, which suggests strong ecological effects of microbiomes during late-stage growth when intraspecific competition intensifies. The *in situ* thermal environment of the seawater used for microbiome assembly influenced host thermal tolerance in a direction opposite to our expectations. All three diatom species reached higher population densities (both as total AUC and maximum density) at the coldest assay (12°C) when paired with microbiomes acquired from the warmest site, San Diego Bay, rather than microbiomes assembled from the coldest site, Kachemak Bay. This trend was consistent at the hottest assay temperature (30°C), where all three species exhibited higher AUC growth, and two species reached higher maximum densities, with microbiomes acquired from Kachemak Bay, rather than San Diego Bay. Although microbiome origin and assay temperature had limited effects on the rate of exponential population growth, *P. tricornutum* showed a response, achieving its highest exponential growth rate at 12°C with a microbiome from San Diego Bay. These findings suggest that microbiomes may have partially detrimental effects on diatom growth, especially during later growth phases once both intraspecific and diatom-bacteria competition has intensified for limiting nutrients. However, we caution that using uniform temperature conditions during the microbiome assembly experiment may have affected this outcome and future work should investigate the functional implications of incubation conditions during assembly. Irrespective of assembly conditions, a mismatch between *in situ* seawater temperature and thermal performance assay temperatures can trigger die-offs of thermally maladaptive microbes, potentially benefitting the host if the loss of pathogenic or competitive microbes outweighed the loss of beneficial microbes. To investigate this potential mechanism, future work should test how varying degrees of thermal mismatch affect bacterial absolute abundance in the diatom microbiome.

Our observation that diatom microbiomes tended to exert a net negative effect on diatom health aligns with certain studies that have shown mostly neutral to antagonistic interactions between cocultured bacterial isolates and marine diatoms [45]. Although most of our results suggest negative impacts of the microbiome on host health, *N. punctata,* which originated from San Diego, tended to outperform non-native diatoms when grown with its local microbiome under thermal conditions matching San Diego Bay. This result aligns with prior research showing that negative effects can be mitigated over time due to coadaptation [45]. These findings highlight the complexity of diatom–bacteria interactions, which may vary depending on host specificity and environmental context. To identify overarching principles that determine when microbiomes confer beneficial versus detrimental effects on host fitness, this work underscores the need to more thoroughly evaluate the roles of both host evolutionary origin and microbiome origin on diatom thermal tolerance.

Beyond the diatom–bacteria system, our results largely diverge from studies where native or local microbiomes have been shown to enhance host fitness. For example, zooplankton hosts coped better with cyanotoxin stress when inoculated with the gut microbiomes from sympatric versus allopatric host populations [25]. Similarly, terrestrial plants were better able to extract limiting resources from their environment when paired with both their local microbial communities and local soil [46]. Given evidence of a home-site advantage of *N. punctata*, future work needs to more thoroughly consider the interactive effects of geographic origin for both hosts and microbiomes in conferring host thermal tolerance.

### Reciprocal microbiome transplants

We found that diatom microbiomes can have host-specific effects on growth, but these effects are strongly context dependent, particularly under thermal stress. The origin of the microbiome, paired with varying degrees of temperature stress, significantly influenced host fitness, as measured by AUC and μ. Specifically, diatoms generally benefitted more from microbiomes derived from different diatom species than their own, particularly under higher levels of temperature stress. This suggests that the host specificity of diatom microbiomes may be driven largely by negative interactions, such as pathogenic effects and competition. By acquiring microbiomes from nonhost species, diatoms may escape these negative interactions, resulting in improved fitness under stress. A caveat to this result is that the diatom strains used in these assays originated from low latitudes; therefore, the microbiomes assembled from the relatively high latitudes of Friday Harbor and Kachemak Bay seawater may have differed substantially from those that coevolved with these diatoms. As both *P. tricornutum* and *N. salinicola* originated from low-latitude environments, neither the “native” nor “non-native” microbiomes used here necessarily reflect those encountered *in situ* under warmer conditions. As such, our use of “native” reflects experimental host specificity rather than ecological origin. These findings expand upon previous studies of phytoplankton–bacteria systems, which primarily focused on the effects of individual bacterial isolates. Such studies consistently documented highly host–species-specific interactions, with bacterial isolates having distinct effects on growth depending on whether the host was their original or nonoriginal partner [45, 47, 48]. Our work now demonstrates the fitness implications of host-specific taxonomically rich microbiomes through reciprocal transplants. However, our prior work with freshwater green algae failed to detect significant host-specific fitness effects, likely because it was conducted under low-stress conditions [29]. These results underscore the role of environmental stress in revealing the fitness consequences of host–microbiome interactions.

Beyond phytoplankton systems, studies in other organisms highlight the fitness benefits conferred by host species-specific microbiomes, particularly under stressful conditions. For example, maternally transmitted microbiomes in dung beetles enhance larval fitness under stress and provide the greatest developmental benefits to their host species [49]. Similarly, the transplants of microbiomes from different congeners within the *Mus* genus into germ-free domestic house mice showed that non-native microbiomes slowed growth and triggered immune responses, further demonstrating the consequences of host-specific microbial associations [50]. Collectively, these findings emphasize the need to determine when and why host-specific microbiomes confer beneficial, neutral, or detrimental effects on host fitness—both within phytoplankton and across broader host–microbiome systems.

Species-specific associations between bacteria and their phytoplankton hosts have been widely documented [48, 51–58], yet whether these patterns of host specificity are consistent across diverse oceanic regions remains uncertain. For example, a study investigated diatom isolates from the North Sea and compared the microbiome composition of two species also used in our study, *D. brightwellii* and *T. pseudonana* [56]. Although this study found that the bacterium *Alteromonas* sp*.* constituted a higher proportion of the microbiome in *D. brightwellii*, we found the opposite pattern with the most common strain of *Alteromonas* averaging 8.1% ± 0.9 SE of the *D. brightwellii* microbiome and 17.2% ± 1.7 SE of the *T. pseudonana* microbiome. Other associations were consistent between studies, such as the predominance of *Marinomonas* spp*.* in association with some diatoms. The study from the North Sea found *Marinomonas* was among the top four most abundant Operational Taxonomic Units (OTUs), whereas we observed several *Marinomonas* strains in abundance with two host species (most common *Marinomonas* OTU comprised 7.2% of the *P. tricornutum* microbiome and 4.5% of the *T. pseudonana* microbiome), yet this genus was rare within the microbiomes of the other four host species studied. Our results also align with broader surveys of marine diatoms and freshwater green algal microbiomes, which frequently report a dominance of *Proteobacteria* [29], although one study found a predominance of *Bacteroidetes* [53]. However, our cultures of *P. tricornutum* were dominated by *γ-Proteobacteria*, rather than the *α-Proteobacteria* commonly found in nonaxenic *P. tricornutum* isolates [59, 60]. Given the reproducibility of γ*-Proteobacteria* colonization across all four of our seawater sites, this result suggests a potential host-specific association with *P. tricornutum* that warrants further study. Future studies could explore whether this dominance persists over time in laboratory cultures or reflects broader ecological dynamics in natural environments.

Our results demonstrate that microbiome assembly is a highly deterministic process driven primarily by host identity, with host species explaining nearly 70% of the variation in microbiome community composition. In contrast, environmental variation—despite spanning a continental gradient across North America—accounts for <10% of this variation. These findings suggest that host microbiome composition, and by extension, its functional role, may remain conserved across steep environmental gradients. However, we caution that our study may have underestimated the effects of environment due to the standardized incubation conditions used during microbiome assembly. Further, although microbiome composition was primarily shaped by host identity rather than the environment, both host-driven and environment-driven variation had significant implications for host fitness. This suggests that host species, even as they shift their geographic ranges in response to climate change, may retain the ability to maintain their core microbiome. However, this interplay between host identity and microbiome composition also reveals vulnerabilities. For instance, variation in host identity at the intraspecific genetic level, which has been shown to regulate microbiome composition and function in other systems [13, 14, 22], could be a key driver of microbiome variation. Shifts in the makeup of host populations—whether through declining genetic diversity or directional selection in response to climate change—could rapidly alter host microbiomes, with cascading effects on host fitness [61]. The potential loss of biodiversity at the intraspecific level may, therefore, have profound implications for microbiome-mediated functions, further compounding the challenges faced by host species under environmental stress. The tight link between host identity and microbiome composition also underscores potential limitations in efforts to manipulate microbiomes for improving health outcomes, such as through dietary interventions or probiotics. These findings extend beyond human health to encompass wildlife conservation and agriculture, where attempts to engineer microbiomes may face similar constraints due to the dominant role of host identity. Nevertheless, although the environment may play a smaller role in shaping microbiome composition, its influence—combined with that of host identity—has quantifiable and substantial implications for host fitness. Understanding this interplay will be key for predicting and mitigating the impacts of environmental change on host–microbiome systems.

## Supplementary Material

Marine_Diatoms_Supplement_4_25_2025_wraf083

## Data Availability

All 16S rRNA sequences have been deposited on QIITA project #15306 and NCBI PRJNA1195978. All other data have been made available through Zenodo at https://zenodo.org/records/15287889
