# Supplementary material for: Host identity drives the assembly of phytoplankton microbiomes across a continental-scale environmental gradient
Source: ISME J. 2025 Apr 30;19(1):wraf083. doi: 10.1093/ismejo/wraf083 (PMC12118458; doi:10.1093/ismejo/wraf083)
Supplement: Marine_Diatoms_Supplement_4_25_2025_wraf083 [file marine_diatoms_supplement_4_25_2025_wraf083.pdf]

Host identity drives the assembly of phytoplankton microbiomes across a continental scale environmental gradient

P. Signe White[2], Taryn Y. Broe[1], Mirte C. M. Kuijpers[1], Jonathan R. Dickey[1] and Sara L. Jackrel[1*]

Supplemental Methods.
Bacterial Amplicon Sequencing and Analysis:

DNA was extracted from half of each 47 mm filter collected during the microbiome assembly and seawater incubation experiments using Qiagen All-Prep kits. Filters were incubated in 600 µL Qiagen RLT buffer with 6 µL 2-mercaptoethanol for 90 minutes on a rotisserie at room temperature. Cell lysis was achieved by vortexing for 10 minutes, followed by homogenization of the lysates using a QIAshredder column. Amplification of 16S rRNA genes was conducted at the UC San Diego Microbiome Core, following protocols established by the Earth Microbiome Project. Briefly, the V4 region of 16S rRNA genes was amplified using Illumina primers with unique forward primer barcodes and the 515fB/806r primer pair. Although these primers are known to amplify mitochondrial and chloroplast sequences from eukaryotic hosts, we opted not to use peptide nucleic acid (PNA) organelle-blocking clamps during PCR amplification. This decision was based on our prior findings that the chloroplast-targeting pPNA sequence biases amplification of nearly 1,500 bacterial taxa commonly found in aquatic environments.(*1, 2*) Amplicons were pooled in equal volumes, and high-throughput sequencing was performed on an MiSeq platform (Illumina) at the UCSD Institute for Genomic Medicine, using paired-end 150 bp cycles. Samples from the seawater incubation experiment were processed as described above, with the exception that library preparation and sequencing was carried out at the Argonne National Laboratory Environmental Sample Preparation & Sequencing Facility.

We processed gene survey data using the QIIME2-dada2 pipeline, with taxonomy assigned using the SILVA 138 reference database.(*3-5*) To ensure our analyses focused on bacterial taxa, we removed all ASVs potentially of eukaryotic origin, including organelle sequences from diatom host cultures. Specifically, we excluded 1,270 ASVs classified as Eukaryotes or unclassified at the Prokaryotic order level, 702 ASVs identified as chloroplast sequences within Cyanobacteria, and 432 ASVs identified as mitochondrial sequences within Rickettsiales. Chloroplast-derived ASVs constituted ~44% of the total reads in the diatom dataset, reducing mean read depth per diatom sample from μ=53,583±12,564 SD to μ=25,742±10,635 SD. Certain diatom microbiome samples, particularly those from *T. pseudonana*, had notably high chloroplast read percentages (μ=82.2±3.9% SD). To address these disparities, we rarefied the dataset at two depths. First, we rarefied all samples to an even depth of 4,995 reads, retaining all six species of diatoms. To ensure that patterns observed at this shallow depth were robust to rarefaction depth, we also rarefied to 19,527 reads, which excluded all samples of *T. pseudonana* due to insufficient read depth. For analyses of individual diatom species, we rarefied to higher library sizes whenever feasible: 10,341 for *A. brevipes*, 22,648 for *D. brightwellii*, 15,671 for *N. punctata*, 21,065 for *N. salinicola*, 5,333 for *T. pseudonana*, and 4,995 for *P. tricornutum*. Rarefaction for each dataset reports relative ASV abundances averaged across 1,000 iterations using the ‘metagMisc’ package.(*6*)

Supplemental References.
1. D. S. Lundberg, S. Yourstone, P. Mieczkowski, C. D. Jones, J. L. Dangl, Practical innovations for high-throughput amplicon sequencing. *Nature Methods* **10**, 999-1002 (2013).

2. S. L. Jackrel, S. M. Owens, J. A. Gilbert, C. A. Pfister, Identifying the plant-associated microbiome across aquatic and terrestrial environments: The effects of amplification method on taxa discovery. *Molecular Ecology Resources* **17**, 931-942 (2017).
3. E. Bolyen *et al.*, Reproducible, interactive, scalable and extensible microbiome data science using QIIME 2. *Nature Biotechnology* **37**, 852-857 (2019).
4. B. J. Callahan *et al.*, DADA2: High-resolution sample inference from Illumina amplicon data. *Nature Methods* **13**, 581-583 (2016).
5. C. Quast *et al.*, The SILVA ribosomal RNA gene database project: Improved data processing and web-based tools. *Nucleic Acids Research* **41**, D590-D596 (2012).
6. V. Mikryukov, metagMisc: Miscellaneous functions for metagenomic analysis. (2019).

Table S1. Summary of temperature parameters for the collection site reported for each phytoplankton strain. GPS coordinates reported for each strain based on database information supplied by the National Center for Marine Algae and Microbiota at Bigelow. Annual temperature data for 2021 (mean ± 1SD) were compiled from NOAA buoys located near each collection site. Note that data is provided for three buoys that surround the collection site for *N. salinicola*. As historical data was not available for the region, we provide 45-day recent averages from Jan. 27 2025 – March 13 2025 for each of these three buoys.

Phytoplankton Species	Strain ID	GPS Coordinates of Strain Collection Site	Location of Collection	NOAA Buoy ID	GPS Coordinates of NOAA Buoy	Mean Temp (µ ± 1 SD)	Temp Range (min - max)
Phaeodactylum tricornutum	CCMP633	13.8°N, 144.8°W	Guam USA	51004	17.5°N, -152.2°W	25.2 ± 0.68	23.8 - 27.4
Navicula salinicola	CCMP1730	16°N, -24°W	Isla do Sol, Cape Verde Islands	13002	21°N, 23°W	22	21.1 - 22.0
Navicula salinicola	CCMP1730	16°N, -24°W	Isla do Sol, Cape Verde Islands	13001	12.0°N, 23°W	24.9	24.4 - 25.4
Navicula salinicola	CCMP1730	16°N, -24°W	Isla do Sol, Cape Verde Islands	13008	15.0°N, 38°W	24.6	23.9 - 25.8
Nitzschia punctata	CCMP2426	32.9°N, -117.3°W	San Diego, California USA	46254- SCRIPPS	32.9°N, -117.3°W	18.0 ± 2.5	13.4 - 25.0
Achnanthes brevipes	CCMP100	41.6°N, -70.6°W	Massachusetts USA	BZBM3- Woods Hole	41.5°N, -70.7°W	13.7 ± 7.5	1.0 - 25.6
Ditylum brightwellii	CCMP3370	47.7°N, -122.4°W	Puget Sound, Washington USA	TCNW1	47.3°N, -122.4°W	11.2 ± 1.7	7.2 - 16.1
Thalassiosira pseudonana	CCMP1015	48.5°N, -123.0°W	Friday Harbor, Washington USA	FRDW1	48.6°N, -123.0°W	9.7 ± 1.4	7.3 - 14.9

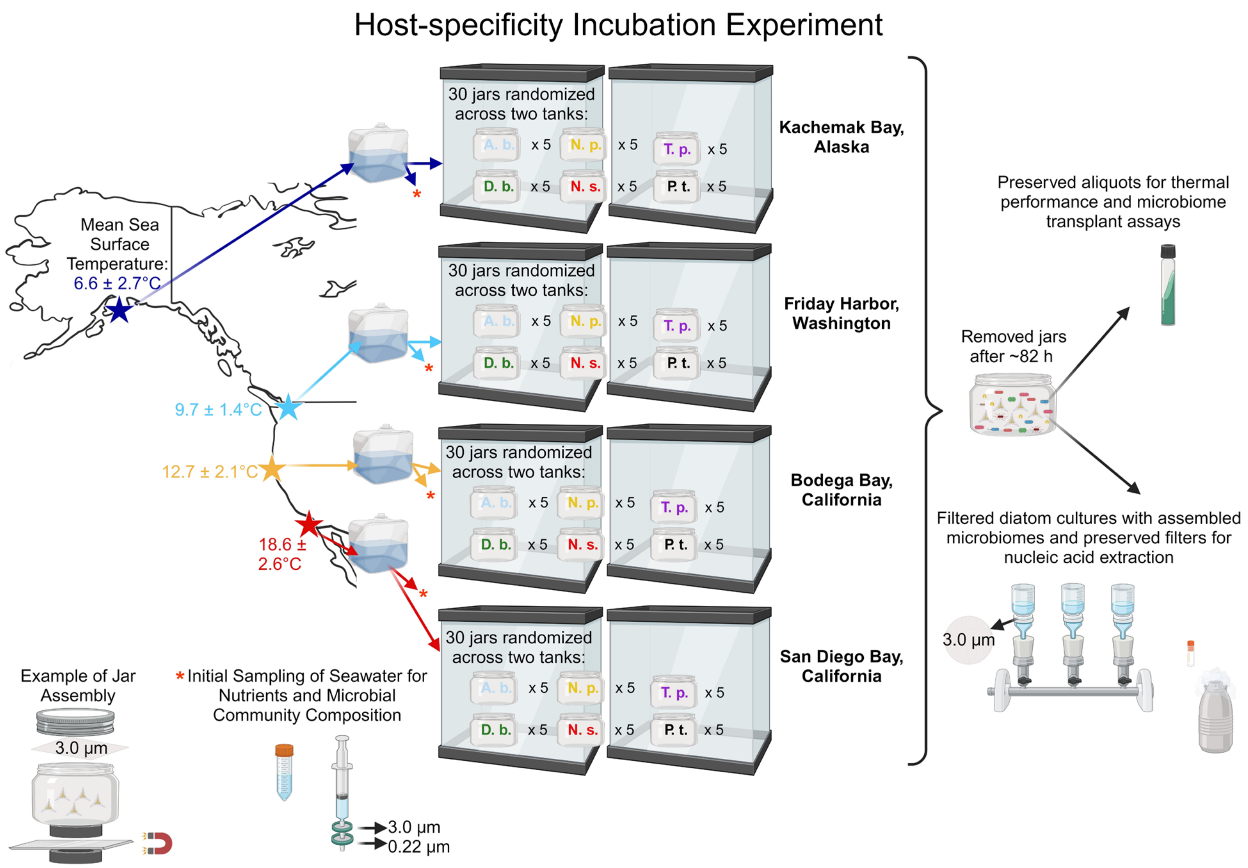

Figure S1. Experimental design for host-specific microbiome incubation study. As an example, at our most northern site, we collected seawater from Kachemak Bay, Alaska across a depth gradient, as described in Table S2. Seawater was immediately transported to our lab space in San Diego, California and poured into two aquarium tanks in which we had jars containing dense monoculture of six species of initially axenic diatoms (A. b. = *A. brevipes*, D. b. = *D. brightwellii*, N. p. = *N. punctata*, N. s. = *N. salinicola*, T. p. = *T. pseudonana*, and P. t. = *P. tricornutum*). After diatoms remained submerged in seawater for ~82 hours to permit microbiome assembly, we preserved biomass from each jar for 16S rRNA sequencing, thermal performance assays and microbiome transplant assays. Illustration generated using BioRender.

Table S2. Summary of water collection sites and environmental parameters. Nutrient concentrations represent the mean ± 1 SD of duplicate measures in seawater samples processed at the Scripps Institution of Oceanography Ocean Data Facility. Salinity was measured once per location using an Ohaus salinity pen. Annual temperature data for 2021 (mean ± 1SD) were compiled from NOAA buoys located near each collection site. Surface temperatures were recorded on-site during water collection, alongside the maximum water collection depth.

Location	GPS Coordinates	NO_3 (µmol/L)	PO_4 (µmol/L)	SIL (µmol/L)	NO_2 (µmol/L)	NH_4 (µmol/l)	Salinity (ppt)	NOAA buoy	Mean Temp (µ ± 1 SD)	Temp Range (min - max)	Surface Temp on Collection Day	Maximum Water Collection Depth (m)
San Diego Bay	32.711139, -117.228417	1.60 ± 0.014	0.48±0.021	10.80± 0.0	0.070±0.0	0.17±0.078	32.4	SDBC1	18.6 ± 2.6	14.2 - 24.8	17.4	5
University of California Bodega Bay Marine Labs	38.323083, -123.054639	1.705 ± 0.092	1.56±0.014	18.10± 0.0	0.18±0.0	4.65±0.23	29.6	BDXC1	12.7 ± 2.1	8.8 - 20.5	12.1	3
University of Washington Friday Harbor Marine Labs	48.545250, -123.012028	22.48±0.14	1.84±0.021	41.05± 0.21	0.37±0.021	0.12±0.057	29.5	FRDW1	9.7 ± 1.4	7.3 - 14.9	10.7	9
Katchemak Bay National Estuarine Research Reserve	59.562306, -151.369694	4.51±0.014	0.66±0.0071	9.35± 0.071	0.095±0.0071	1.66±0.035	32.1	OVIA2	6.6 ± 2.7	1 - 12.2	5.8	20

Supplemental Results.

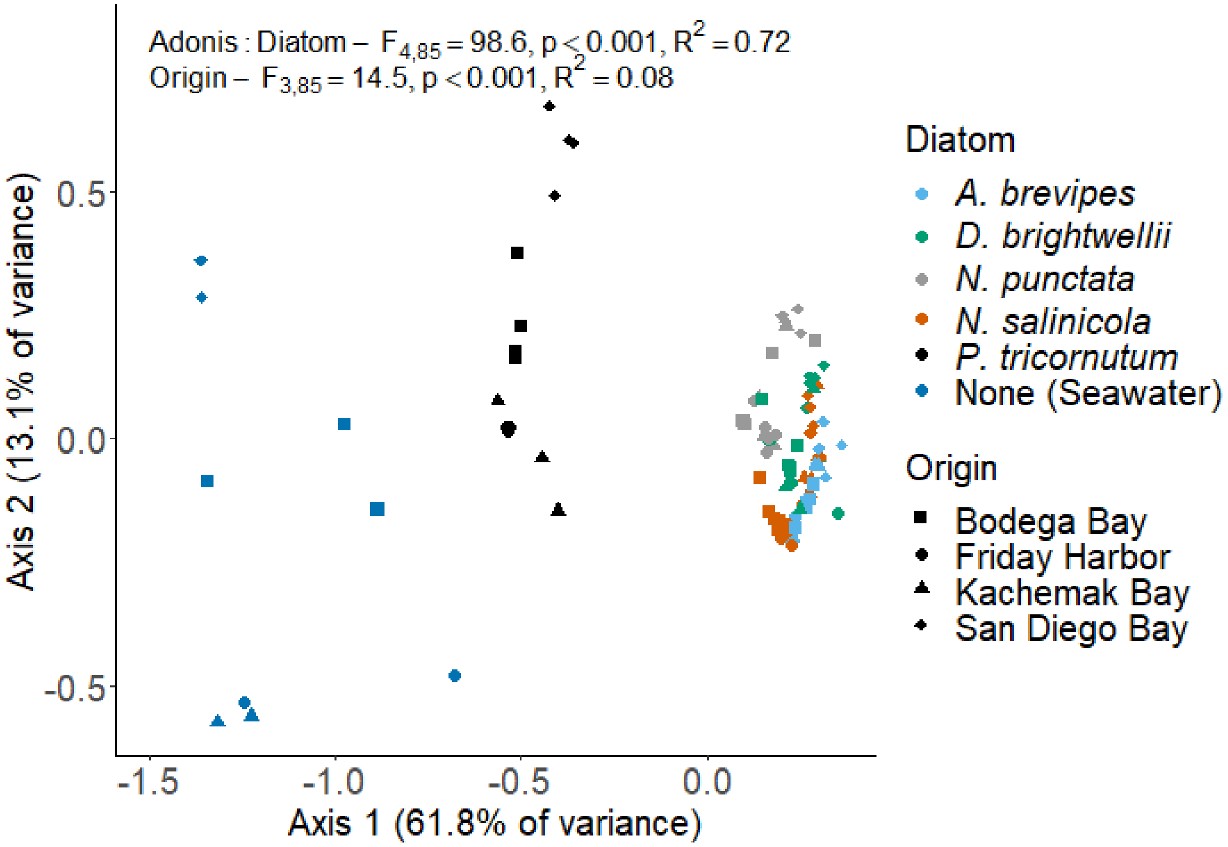

Figure S2. Host-specific microbiome assembly is robust across rarefaction depths. Patterns of host species specificity following an 82 hour incubation period of initially axenic marine diatoms in seawater remain consistent across rarefaction depths. Shown here are bacterial community compositional differences using a Bray-Curtis distance metric with a rarefaction depth of 19,527, which aligns closely with patterns observed at a rarefaction depth of 4,995 (as seen Fig. 1). Seawater used for this experiment was collected along a continental-scale thermal gradient, spanning from San Diego Bay, California in the South (32.7°N) to Kachemak Bay, Alaska (59.6°N). Significant bacterial compositional differences were observed among diatom species (Adonis: Diatom $F_{4,85}$=98.6, p<0.001, R^2=0.72; pairwise post hoc tests for all diatom species, p<0.001). Seawater samples, included in the ordination for visual comparison, were excluded from statistical analyses to focus solely on differences among diatom species. The ordination highlights host-driven microbiome community structure, robust to differences in rarefaction depth.

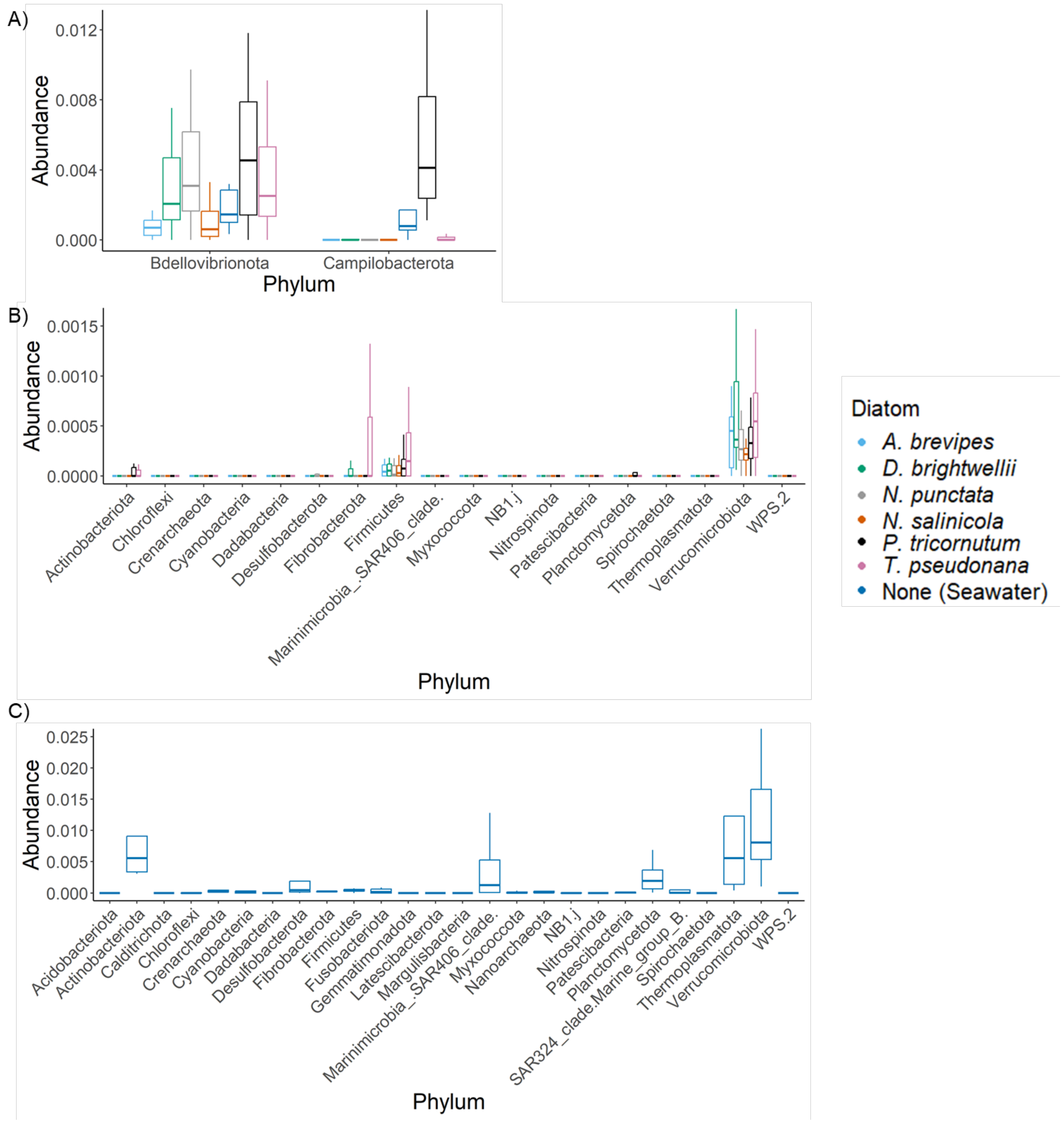

Figure S3. Relative abundances of less dominant microbial phyla in diatom microbiomes. Mean proportional abundances of less dominant phyla associated with the microbiomes of six marine diatom species and surrounding seawater. A) Phyla that constitute 1-10% of microbiomes across diatom species and seawater samples. B) Phyla contributing less than 1% of the diatom microbiome. C) Phyla contributing less than 1% of the microbial communities in seawater. See Fig. 1B for the three most abundant phyla in diatom microbiomes and seawater. Data are presented as box plots to visualize variability within each category.

Table S3. Relative abundances of common ASVs in diatom microbiomes. Relative abundances of Amplicon Sequence Variants (ASVs) recruited by six species of initially axenic diatom hosts from the surrounding seawater. This tables includes only common ASVs, defined as taxa that constitute at least 1% of the microbiome in at least one diatom microbiome sample. ASVs are listed alongside their taxonomic classifications and mean relative abundances (± standard error) are provided for each diatom species and seawater.

Seawater		*A. brevipes*		*D. brightwellii*		*N. punctata*		*N. salinicola*		*P. tricornutum*		*T. pseudonana*		Taxonomic Classification
μ	S.E.	μ	S.E.	μ	S.E.	μ	S.E.	μ	S.E.	μ	S.E.	μ	S.E.	
0.151	0.076	0.289	0.071	0.910	0.359	1.478	0.378	0.224	0.038	27.786	3.785	8.137	1.654	Gammaproteobacteria;Vibrionales;Vibrionaceae
0.001	0.001	45.678	1.952	28.819	2.051	11.660	0.824	36.197	2.201	0.286	0.069	14.456	1.281	Alphaproteobacteria;Caulobacterales;Hyphomonadaceae;Oceanicaulis spp.
0.009	0.004	12.971	0.956	36.526	2.178	23.908	1.442	6.182	0.536	0.513	0.194	13.670	1.026	Alphaproteobacteria;Rhodobacterales;Rhodobacteraceae;Roseobacter spp.
0.002	0.002	9.154	0.913	8.121	0.936	21.879	2.241	10.115	0.925	3.908	0.635	17.218	1.670	Gammaproteobacteria;Alteromonadales;Alteromonadaceae;Alteromonas spp.
0.060	0.038	0.395	0.080	0.933	0.367	1.222	0.252	0.547	0.180	22.234	2.607	6.794	1.014	Gammaproteobacteria;Vibrionales;Vibrionaceae;Vibrio spp.
0.000	0.000	0.323	0.032	2.993	0.571	13.034	2.057	0.673	0.057	0.062	0.016	3.734	0.408	Alphaproteobacteria;Rhodobacterales;Rhodobacteraceae;Roseovarius spp.
0.056	0.026	1.620	0.651	0.652	0.375	0.035	0.013	3.672	2.048	0.008	0.005	0.324	0.232	Bacteroidota;Bacteroidia;Flavobacteriales;NS9 marine group;Flavobacterium spp.
0.000	0.000	14.426	1.555	2.474	0.363	10.751	0.694	21.915	1.522	0.137	0.036	6.680	0.832	Alphaproteobacteria;Rhodobacterales;Rhodobacteraceae
0.043	0.017	0.054	0.012	0.177	0.051	1.072	0.426	0.057	0.021	7.153	2.499	4.538	1.320	Gammaproteobacteria;Oceanospirillales;Marinomonadaceae;Marinomonas spp.
1.007	0.385	7.667	1.500	8.550	1.629	4.419	0.907	10.674	1.886	2.714	0.474	9.765	1.643	Bacteroidota;Bacteroidia;Flavobacteriales;Cryomorphaceae;Flavobacteriia spp.
0.005	0.004	0.046	0.032	0.425	0.409	0.004	0.002	0.311	0.301	1.188	1.048	0.006	0.004	Gammaproteobacteria;Alteromonadales;Pseudoalteromonadaceae;Algicola bacteriolytica
0.017	0.012	0.137	0.068	0.013	0.005	0.169	0.094	0.148	0.088	4.358	1.663	1.552	0.637	Gammaproteobacteria;Oceanospirillales;Marinomonadaceae;Marinomonas spp.
2.677	1.105	0.064	0.018	0.122	0.073	0.069	0.014	0.137	0.046	5.660	1.033	0.242	0.060	Gammaproteobacteria;Alteromonadales;Alteromonadaceae;Glaciecola spp.
0.528	0.221	0.401	0.107	2.290	0.781	2.704	0.833	0.616	0.253	1.400	0.445	3.467	0.795	Bacteroidota;Bacteroidia;Flavobacteriales;Cryomorphaceae
0.151	0.103	1.117	0.339	0.612	0.172	0.117	0.047	3.131	0.888	0.042	0.017	0.156	0.070	Bacteroidota;Bacteroidia;Flavobacteriales;Crocinitomicaceae;Crocinitomix spp.
0.058	0.038	0.007	0.004	0.197	0.114	1.671	0.606	0.006	0.002	0.383	0.229	1.224	0.560	Gammaproteobacteria;Alteromonadales;Pseudoalteromonadaceae;Psychrosphaera spp.
0.000	0.000	0.000	0.000	0.002	0.002	0.038	0.022	0.000	0.000	1.258	0.523	0.312	0.146	Gammaproteobacteria;Oceanospirillales;Marinomonadaceae;Marinomonas spp.
0.018	0.012	0.003	0.002	0.048	0.038	0.027	0.013	0.004	0.002	1.233	0.478	0.081	0.033	Gammaproteobacteria;Oceanospirillales;Marinomonadaceae;Marinomonas spp.
0.000	0.000	2.504	0.252	0.004	0.002	0.014	0.007	0.535	0.061	0.002	0.001	0.001	0.001	Alphaproteobacteria;Rhodobacterales;Rhodobacteraceae
0.004	0.003	0.009	0.005	0.008	0.004	0.332	0.300	0.014	0.006	0.075	0.033	0.021	0.010	Gammaproteobacteria;Cellvibrionales;Cellvibrionaceae;Marinagarivorans spp.
0.000	0.000	0.248	0.248	0.000	0.000	0.001	0.001	0.037	0.037	0.000	0.000	0.000	0.000	Bacteroidota;Bacteroidia;Flavobacteriales;Cryomorphaceae
0.000	0.000	0.000	0.000	0.001	0.001	0.025	0.011	0.001	0.001	0.918	0.365	0.172	0.069	Gammaproteobacteria;Oceanospirillales;Marinomonadaceae;Marinomonas spp.
1.654	0.363	0.000	0.000	0.002	0.002	0.006	0.004	0.000	0.000	1.390	0.330	0.050	0.017	Alphaproteobacteria;Rhodobacterales;Rhodobacteraceae;Sulfitobacter spp.
0.000	0.000	0.000	0.000	0.001	0.001	0.000	0.000	0.000	0.000	0.205	0.203	0.000	0.000	Gammaproteobacteria;Alteromonadales;Pseudoalteromonadaceae;Algicola bacteriolytica
0.013	0.013	0.000	0.000	0.000	0.000	0.234	0.103	0.000	0.000	0.494	0.247	0.061	0.045	Bacteroidota;Bacteroidia;Flavobacteriales;Cryomorphaceae
0.056	0.053	0.214	0.090	0.108	0.056	0.033	0.021	0.207	0.193	0.039	0.028	0.201	0.135	Bacteroidota;Bacteroidia;Flavobacteriales;Crocinitomicaceae;Fluviicola spp.
0.001	0.001	0.001	0.001	0.013	0.009	0.062	0.039	0.007	0.006	0.774	0.307	0.511	0.250	Gammaproteobacteria;Oceanospirillales;Marinomonadaceae;Marinomonas spp.
0.000	0.000	0.000	0.000	0.001	0.001	0.000	0.000	0.001	0.001	0.364	0.188	0.005	0.004	Campilobacterota;Campylobacteria;Campylobacterales;Arcobacteraceae
0.001	0.001	0.001	0.001	0.000	0.000	0.001	0.000	0.001	0.001	0.459	0.174	0.004	0.002	Gammaproteobacteria;Oceanospirillales;Nitrincolaceae;Neptuniibacter spp.
0.000	0.000	0.011	0.003	0.024	0.009	0.280	0.172	0.005	0.002	0.076	0.032	0.031	0.015	Bdellovibrionota;Bdellovibrionia;Bacteriovoracales;Bacteriovoracaceae;Halobacteriovorax spp.
0.000	0.000	0.010	0.003	0.965	0.141	0.075	0.013	0.339	0.041	0.075	0.018	0.106	0.014	Gammaproteobacteria;Alteromonadales;Marinobacteraceae;Marinobacter spp.
0.000	0.000	0.600	0.051	1.453	0.146	0.018	0.009	1.422	0.121	0.020	0.007	0.346	0.046	Alphaproteobacteria;Rhodobacterales;Rhodobacteraceae
0.000	0.000	0.014	0.005	0.082	0.033	0.011	0.005	0.140	0.065	0.096	0.040	0.290	0.146	Bdellovibrionota;Bdellovibrionia;Bacteriovoracales;Bacteriovoracaceae;Peredibacter spp.
0.116	0.091	0.000	0.000	0.005	0.004	0.003	0.003	0.000	0.000	0.413	0.178	0.004	0.004	Gammaproteobacteria;Alteromonadales;Pseudoalteromonadaceae;Pseudoalteromonas spp.
0.084	0.080	0.020	0.015	0.143	0.132	0.004	0.002	0.002	0.001	0.008	0.007	0.004	0.002	Bacteroidota;Bacteroidia;Chitinophagales;Saprospiraceae
0.028	0.013	0.000	0.000	0.000	0.000	0.000	0.000	0.001	0.001	0.374	0.170	0.004	0.004	Campilobacterota;Campylobacteria;Campylobacterales;Arcobacteraceae
0.531	0.528	0.031	0.017	0.040	0.022	0.043	0.030	0.005	0.003	0.116	0.100	0.134	0.113	Bacteroidota;Bacteroidia;Flavobacteriales;Cryomorphaceae
0.000	0.000	0.105	0.105	0.000	0.000	0.000	0.000	0.000	0.000	0.000	0.000	0.000	0.000	Bacteroidota;Bacteroidia;Flavobacteriales;Flavobacteriaceae;Flavicella spp.
0.000	0.000	0.002	0.001	0.036	0.019	0.260	0.140	0.003	0.001	0.011	0.006	0.020	0.011	Bdellovibrionota;Bdellovibrionia;Bacteriovoracales;Bacteriovoracaceae
1.681	1.134	0.005	0.003	0.004	0.003	0.010	0.006	0.000	0.000	0.294	0.133	0.062	0.032	Alphaproteobacteria;Rhodobacterales;Rhodobacteraceae;HIMB11 spp.
0.150	0.071	0.001	0.001	0.000	0.000	0.003	0.002	0.001	0.001	0.290	0.124	0.010	0.004	Gammaproteobacteria;Oceanospirillales;Nitrincolaceae;Marinobacterium spp.
1.124	0.455	0.163	0.034	0.090	0.020	0.055	0.012	0.306	0.061	0.692	0.110	0.270	0.067	Bacteroidota;Bacteroidia;Flavobacteriales;Flavobacteriaceae;NS3a marine group
0.000	0.000	0.014	0.003	0.006	0.001	0.944	0.076	0.269	0.022	0.003	0.002	0.572	0.047	Alphaproteobacteria;Rhodobacterales;Rhodobacteraceae
0.006	0.003	0.000	0.000	0.075	0.053	0.250	0.116	0.000	0.000	0.091	0.051	0.014	0.008	Gammaproteobacteria;Alteromonadales;Colwelliaceae;Thalassotalea spp.
0.001	0.001	0.001	0.001	0.001	0.001	0.204	0.104	0.001	0.001	0.235	0.105	0.002	0.001	Gammaproteobacteria;Alteromonadales;Pseudoalteromonadaceae;Psychrosphaera spp.
0.005	0.002	0.001	0.001	0.001	0.000	0.000	0.000	0.000	0.000	0.194	0.086	0.002	0.001	Campilobacterota;Campylobacteria;Campylobacterales;Arcobacteraceae
0.000	0.000	0.000	0.000	0.475	0.068	0.032	0.010	0.157	0.022	0.032	0.008	0.038	0.011	Gammaproteobacteria;Alteromonadales;Marinobacteraceae;Marinobacter spp.
0.000	0.000	0.005	0.003	0.002	0.002	0.014	0.009	0.007	0.004	0.169	0.082	0.041	0.018	Gammaproteobacteria;Vibrionales;Vibrionaceae;Vibrio spp.
1.986	0.462	0.051	0.010	0.070	0.013	0.129	0.031	0.010	0.003	0.448	0.066	0.235	0.070	Gammaproteobacteria;Oceanospirillales;Pseudohongiellaceae;Pseudohongiella spp.
0.000	0.000	0.004	0.003	0.018	0.018	0.000	0.000	0.015	0.014	0.087	0.071	0.001	0.001	Gammaproteobacteria;Alteromonadales;Pseudoalteromonadaceae;Algicola bacteriolytica
0.143	0.054	0.001	0.001	0.002	0.001	0.001	0.001	0.001	0.000	0.244	0.081	0.002	0.002	Alphaproteobacteria;Rhodobacterales;Rhodobacteraceae
0.002	0.002	0.085	0.070	0.021	0.017	0.001	0.001	0.017	0.011	0.000	0.000	0.006	0.004	Bacteroidota;Bacteroidia;Flavobacteriales;Cryomorphaceae;Owenweeksia spp.
0.000	0.000	0.006	0.002	0.016	0.004	0.147	0.078	0.005	0.002	0.095	0.033	0.034	0.015	Bdellovibrionota;Bdellovibrionia;Bacteriovoracales;Bacteriovoracaceae;Halobacteriovorax spp.
0.033	0.015	0.000	0.000	0.006	0.005	0.103	0.044	0.000	0.000	0.213	0.080	0.065	0.049	Gammaproteobacteria;Alteromonadales;Pseudoalteromonadaceae;Psychrosphaera spp.
0.062	0.038	0.027	0.008	0.038	0.016	0.043	0.019	0.036	0.009	0.238	0.068	0.071	0.034	Gammaproteobacteria;Oceanospirillales;Saccharospirillaceae;Oceaniserpentilla haliotis
0.788	0.490	0.018	0.007	0.013	0.006	0.004	0.002	0.171	0.069	0.041	0.014	0.003	0.002	Bacteroidota;Bacteroidia;Flavobacteriales;Flavobacteriaceae;Polaribacter spp.
#####	3.020	0.013	0.003	0.015	0.005	0.042	0.010	0.010	0.003	0.490	0.077	0.167	0.033	Alphaproteobacteria;Rhodobacterales;Rhodobacteraceae;Amylibacter spp.
0.377	0.236	0.000	0.000	0.118	0.072	0.002	0.001	0.000	0.000	0.000	0.000	0.000	0.000	Verrucomicrobiota;Verrucomicrobiae;Verrucomicrobiales;Rubritaleaceae;Persicirhabdus spp.
0.580	0.292	0.003	0.002	0.001	0.001	0.006	0.003	0.002	0.001	0.178	0.074	0.016	0.008	Alphaproteobacteria;Rhodobacterales;Rhodobacteraceae
0.017	0.016	0.000	0.000	0.057	0.057	0.000	0.000	0.001	0.001	0.042	0.011	0.000	0.000	Gammaproteobacteria;Alteromonadales;Alteromonadaceae;Alteromonas spp.
0.124	0.084	0.012	0.004	0.019	0.005	0.079	0.032	0.015	0.005	0.193	0.063	0.049	0.018	Gammaproteobacteria;Oceanospirillales;Saccharospirillaceae
0.000	0.000	0.000	0.000	0.000	0.000	0.001	0.001	0.000	0.000	0.052	0.051	0.029	0.014	Gammaproteobacteria;Oceanospirillales;Marinomonadaceae;Marinomonas spp.
0.000	0.000	0.000	0.000	0.000	0.000	0.000	0.000	0.001	0.001	0.158	0.068	0.000	0.000	Campilobacterota;Campylobacteria;Campylobacterales;Arcobacteraceae

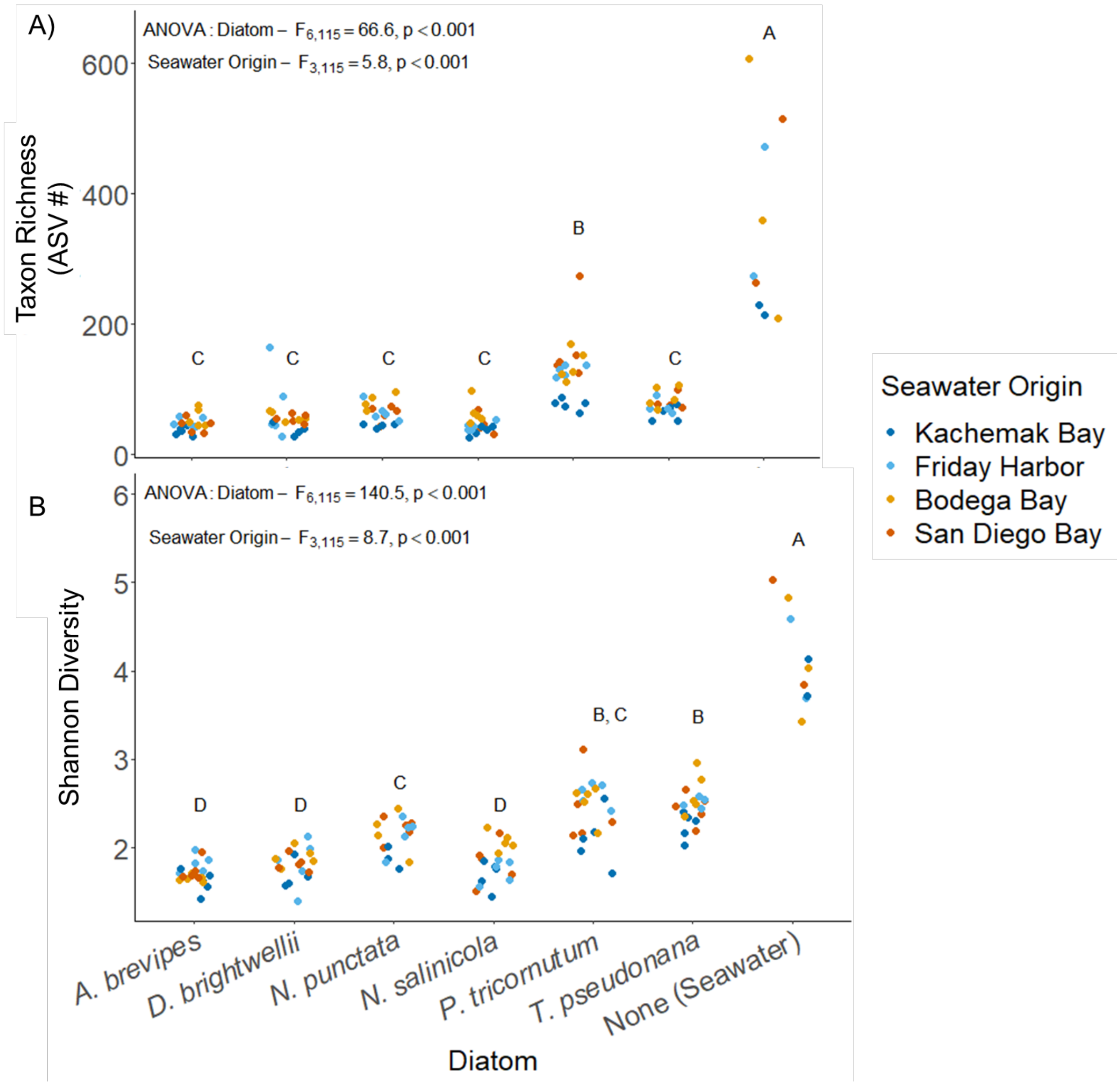

Figure S4. Host identity and the environment shape microbiome richness and diversity. Microbiomes assembled by initially axenic diatoms from seawater exhibited significant variation in A) taxon richness and B) Shannon diversity both across diatom species and environments. Pairwise post hoc tests revealed significant differences in bacterial richness and diversity among diatom species (species with different capital letters are significantly different at *p*<0.05). Both taxon richness and Shannon diversity were significantly lower in diatom microbiomes compared to the seawater from which they were assembled. Among seawater origins, microbiomes assembled from Kachemak Bay consistently showed significantly lower richness and diversity compared to microbiomes assembled from the three other sites (San Diego Bay, Bodega Bay and Friday Harbor), which exhibited similar values.

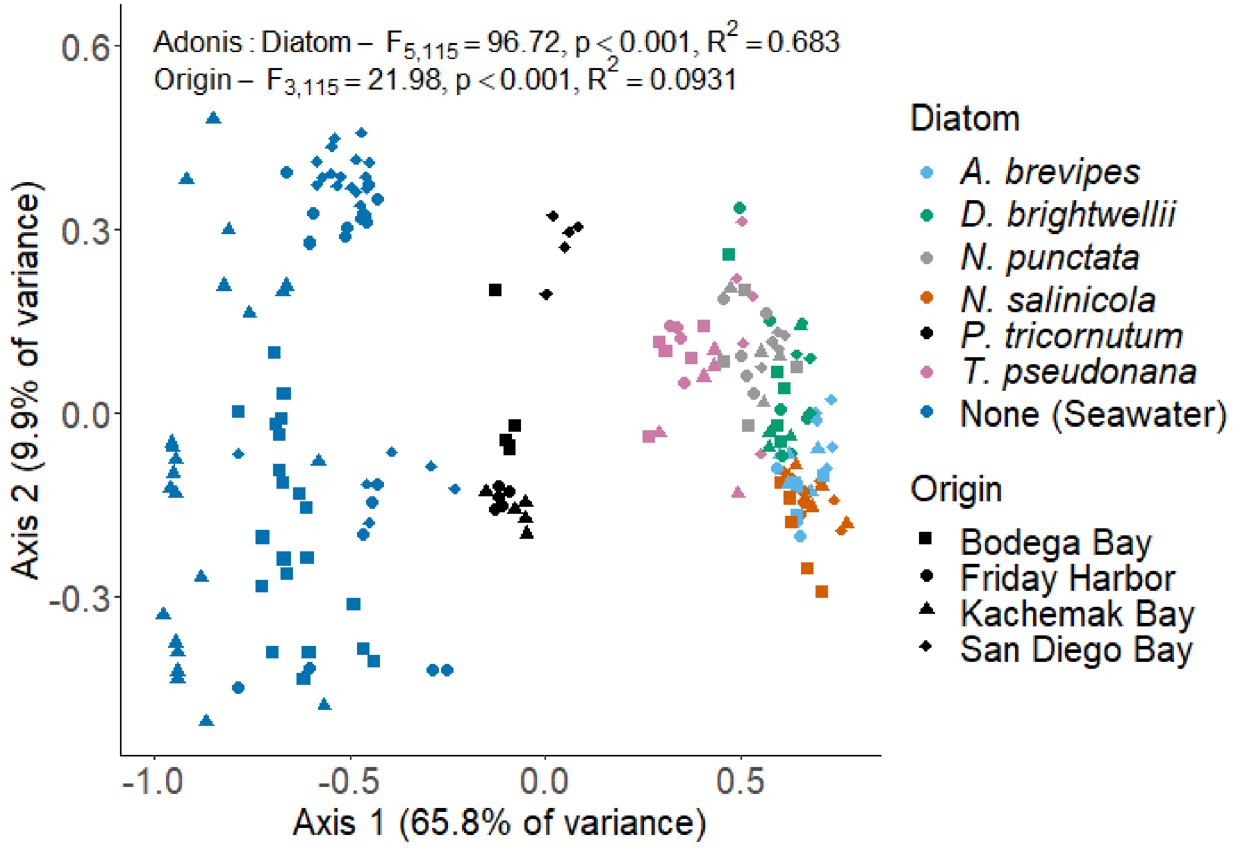

Figure S5. Host-specific microbiomes differ from seawater microbial communities. Host-specific microbiomes assembled after an 82-hour incubation period were distinct from all seawater samples. These seawater samples included those collected at T_0 of the main study as well as samples collected after 0, 24, 48, 72 and 82 hours during a secondary incubation experiment. This follow-up study was specifically designed to assess any shifts in bacterial community composition over time under the controlled temperature and lighting conditions of the original incubation experiment.

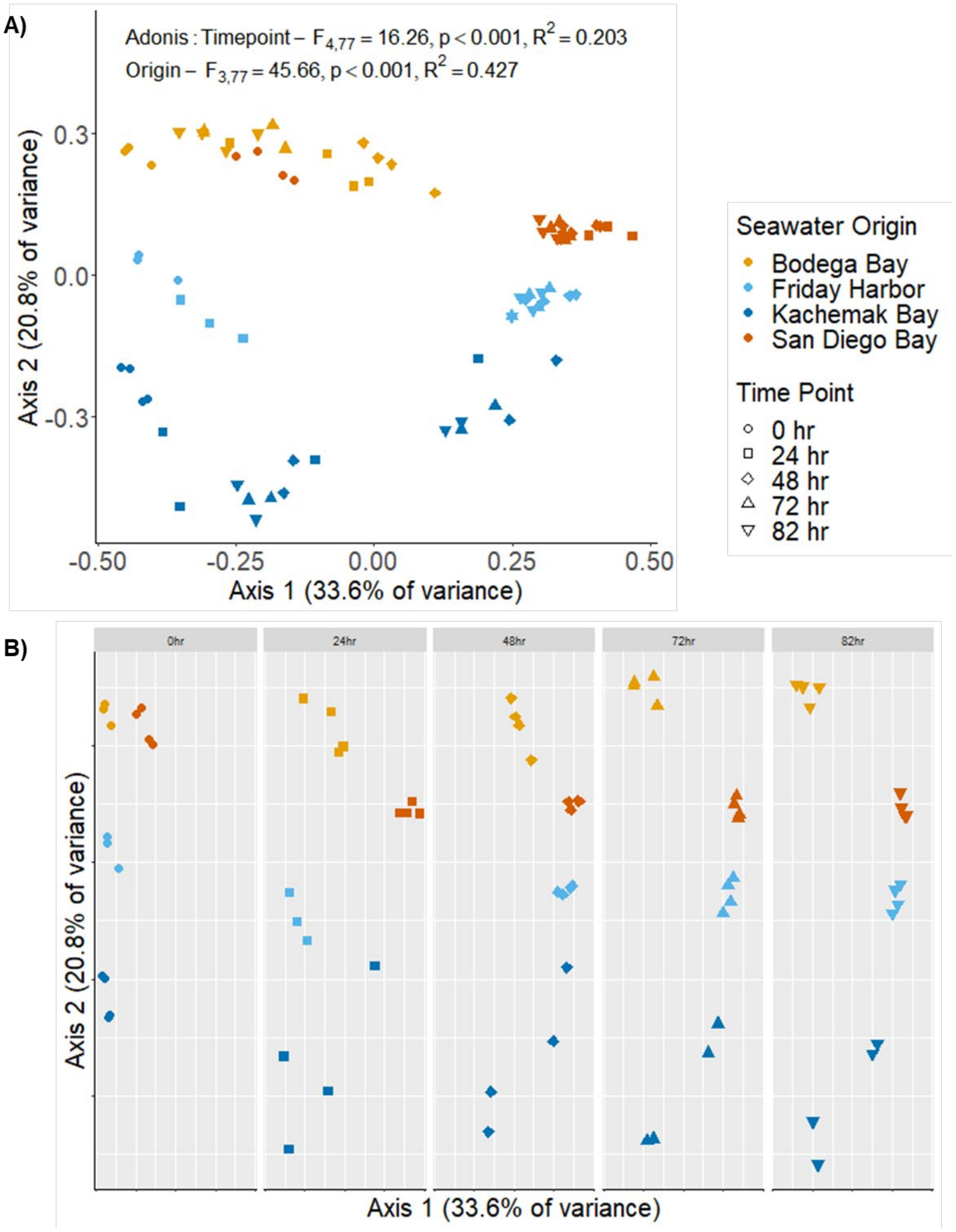

Figure S6. Seawater origin dominates bacterial compositional differences over time. Seawater origin was the primary determinant of bacterial community composition, while incubation time played a secondary but significant role. Bacterial communities differed significantly among all seawater origins (pairwise post-hoc comparisons, p<0.001). While significant differences in bacterial composition were observed between the initial timepoint (0 h) and all subsequent timepoints (pairwise comparisons, p-values<0.01), community composition stabilized from 24 h to 82h (pairwise comparisons, p-values>0.05). Panel A highlights the persistent influence of seawater origin on bacterial community composition across all timepoints. Panel B shows the distinct clustering of bacterial communities by seawater origin, independent of incubation time.

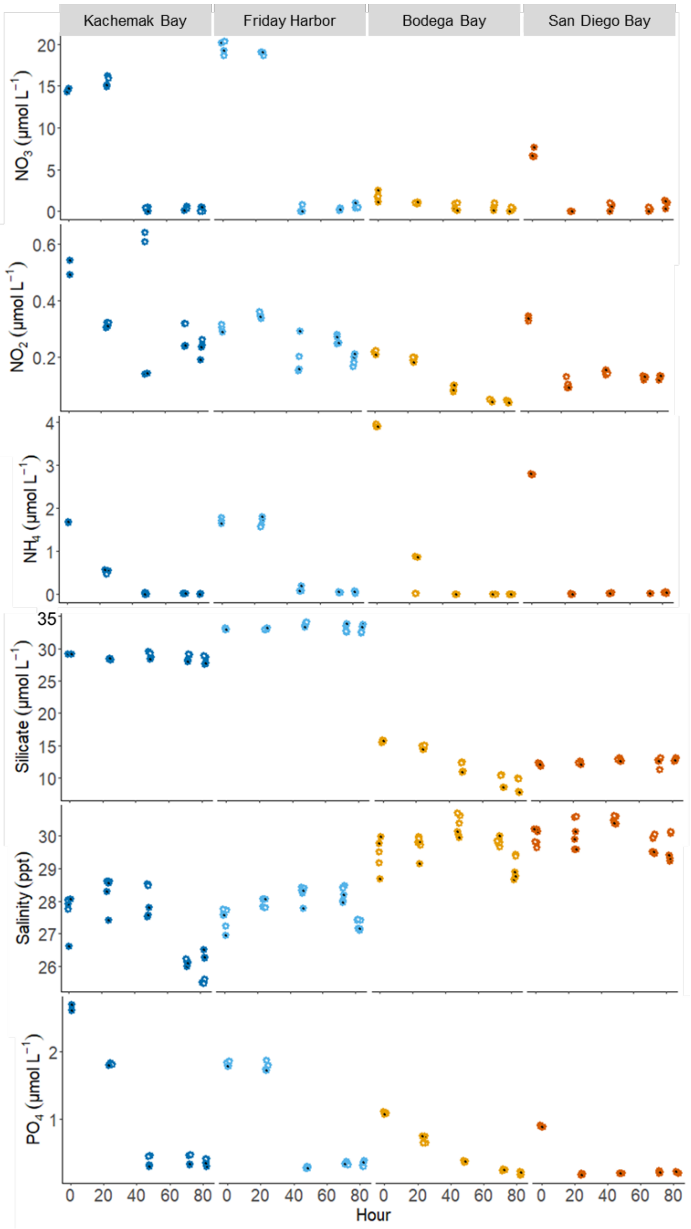

Figure S7. Nutrient variation during seawater incubation study. Nutrient concentrations during the seawater incubation study conducted in April of 2024 were monitored over time, aligned with the amplicon sequencing timeline. Each location had two aquarium tanks, represented by white and black fills. Salinity measurements were taken in triplicate with an Ohaus salinity pen, while all other nutrient concentrations were measured in duplicate and analyzed at the Scripps Institution of Oceanography Ocean Data Facility. Significant differences in nutrient concentrations were observed across timepoints and seawater origins for most metrics: **NO_3** (timepoint: $F_{1,70}$=136.6, $p<0.01$, seawater origin $F_{3,70}$=24.5, $p<0.01$), **NO_2** (timepoint: $F_{1,73}$=40.9, $p<0.01$, origin: $F_{3,73}$=29.7, $p<0.01$), **NH_4** (timepoint: $F_{1,70}$=118.3, $p<0.01$, origin: $F_{3,70}$=1.4, $p=0.26$), **silicate** (timepoint: $F_{1,70}$=33.4, $p<0.01$, origin: $F_{3,70}$=6413.3, $p<0.01$), **salinity** (timepoint: $F_{1,112}$=20.0, $p<0.01$, origin: $F_{3,112}$=178.7, $p<0.01$), **PO_4** (timepoint: $F_{1,70}$=213.8, $p<0.01$, origin: $F_{3,70}$=26.7, $p<0.01$).

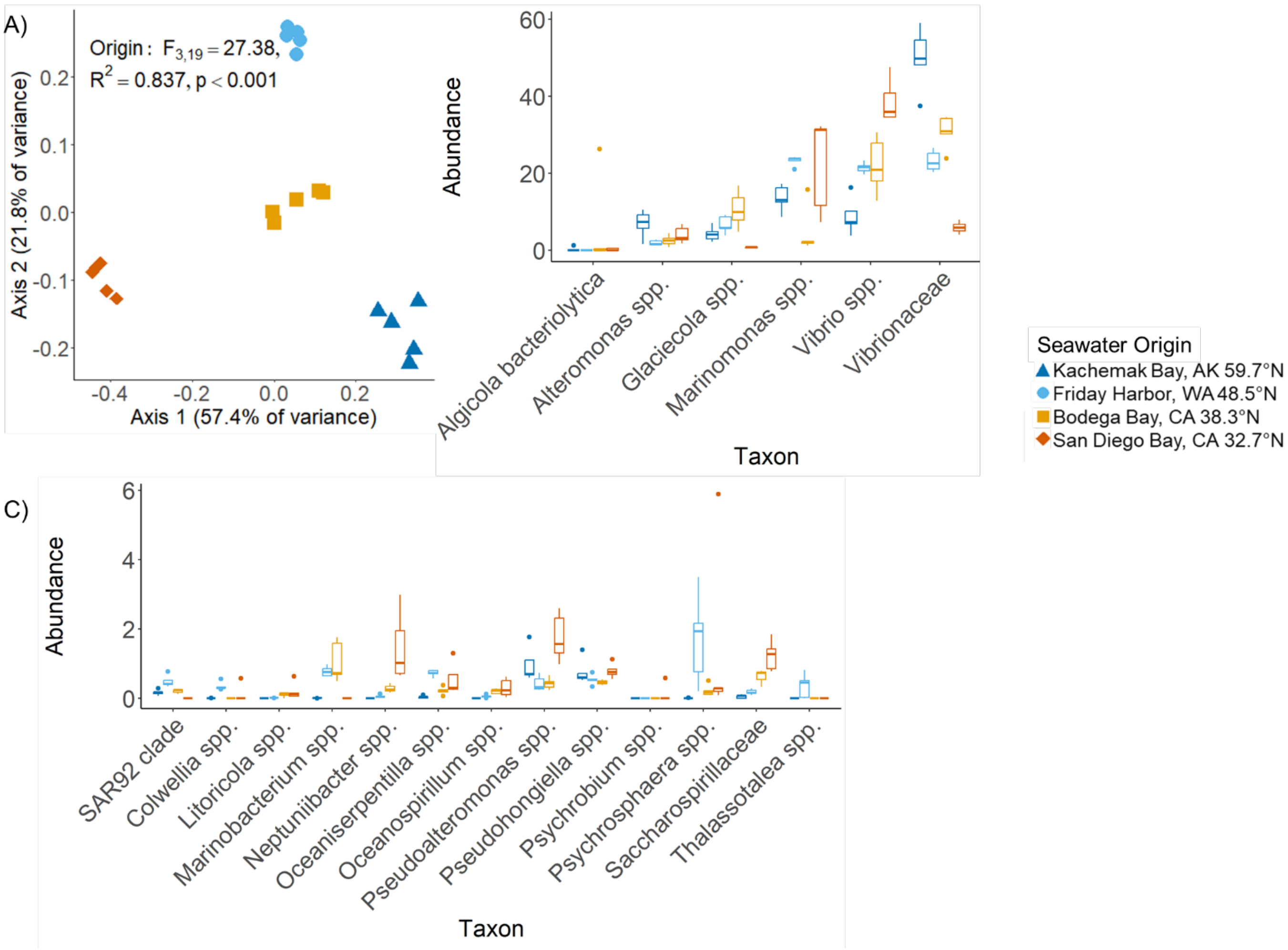

Figure S8. γ-Proteobacteria dominate *P. tricornutum* microbiomes across locations. A) Within cultures of initially axenic *P. tricornutum*, the predominant bacterial phyla assembled from seawater following the incubation was γ-Proteobacteria. Bacterial community composition within the γ-Proteobacteria, visualized using a Bray-Curtis distance metric, exhibited significant divergence across the four seawater collection sites spanning a continental-scale gradient. B) Mean proportional abundances of common γ-Proteobacteria in the *P. tricornutum* microbiome assembled after incubation in seawater. C) Mean proportional abundances of rare γ-Proteobacteria in the *P. tricornutum* microbiome. Taxa are shown if they constituted at least 0.5% abundance in at least one sample.

Table S4. Home-site advantage of local diatom species. To determine whether local adaptation of the diatom host may have contributed to observed fitness measures, we tested whether *N. punctata* tended to outperform when paired with a local microbiome and local temperature conditions, relative to non-local diatom species. All area under the curve measures per diatom species were converted to z-scores to standardize for differences in growth among species. Standardized AUC values are reported for each species grown with its microbiomes acquired from San Diego Bay. *Nitzschia punctata* tended to outperform compared to non-native diatoms at temperature conditions that have been observed in San Diego Bay: 18 and 24°C. These trends were not observed at temperatures that were colder or warmer than the minimum and maximum values observed in San Diego Bay. One-way ANOVA models test whether standardized AUC values of the Native versus Non-Native hosts statistically differed. Also shown are results for standardized exponential rate of growth and standardized maximum density.

	Standardized AUC (mean)			
	Native Host	Non-Native Hosts		
Temperature (°C)	*N. punctata*	*N. salinicola*	*P. tricornutum*	ANOVA
12	-0.450	-0.836	-0.104	$F_{1,6} = 0.01$, $p = 0.92$
**18**	**-0.379**	**-0.910**	**-1.005**	$\mathbf{F_{1,7} = 12.52}$, $\mathbf{p = 0.012}$
24	0.197	-0.333	-0.183	$F_{1,6} = 1.66$, $p = 0.25$
30	-0.711	1.506	-0.571	$F_{1,4} = 0.69$, $p = 0.47$

	Standardized μ (mean)			
	Native Host	Non-Native Hosts		
Temperature (°C)	*N. punctata*	*N. salinicola*	*P. tricornutum*	ANOVA
12	0.348	0.279	0.601	$F_{1,6} = 0.02$, $p = 0.89$
18	-0.345	0.672	-0.385	$F_{1,7} = 0.60$, $p = 0.47$
24	-0.231	-0.622	-0.622	$F_{1,5} = 0.10$, $p = 0.76$
30	-0.141	-0.262	-0.262	$F_{1,4} = 0.87$, $p = 0.42$

	Standardized Maximum Density (mean)			
	Native Host	Non-Native Hosts		
Temperature (°C)	*N. punctata*	*N. salinicola*	*P. tricornutum*	ANOVA
12	-0.662	-0.750	-0.164	$F_{1,6} = 0.25$, $p = 0.64$
**18**	**-0.413**	**-0.879**	**-1.257**	$\mathbf{F_{1,7} = 8.43}$, $\mathbf{p = 0.027}$
24	0.418	0.168	0.049	$F_{1,6} =0.26$, $p = 0.63$
30	-0.312	1.686	0.097	$F_{1,4} = 0.84$, $p = 0.43$

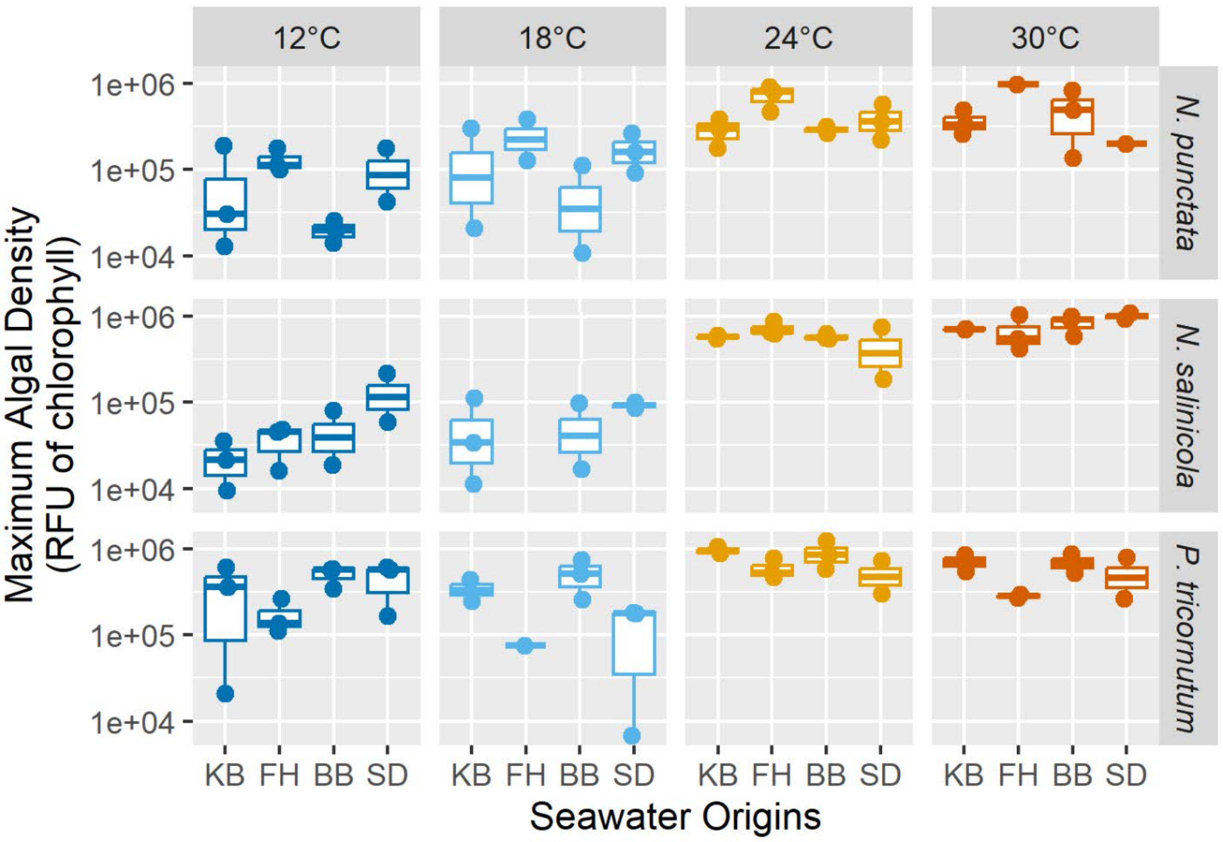

Figure S9. Temperature and microbiome origin influence diatom growth densities. The origin of the microbiome and incubation temperature significantly influenced the maximum algal density achieved by diatom hosts. Maximum density (measured as the highest RFU value of chlorophyll-a fluorescence across the growth curve, see Fig. S11) was recorded for three diatom species grown with microbiomes assembled from one of four seawater sources: KB = Kachemak Bay, Alaska; FH = Friday Harbor, Washington; BB = Bodega Bay, California; SD = San Diego Bay, California. Cultures were incubated in L1 media at one of four temperatures for 29 days. Two-way ANOVA tests assessed the effects of Temperature and Seawater Origin as fixed effects, excluding the interaction term when $p>0.05$. Results are as follows: *Nitzschia punctata*: Temperature- $F_{3,32}=12.24$, $p<0.01$, Seawater Origin- $F_{3,32}=5.04$, $p<0.01$. *Navicula salinicola*: Temperature- $F_{3,31}=39.84$, $p<0.01$, Origin - $F_{3,31}=0.57$, $p=0.64$. *Phaeodactylum tricornutum*: Temperature- $F_{3,34}=11.15$, $p<0.01$, Origin- $F_{3,34}=6.57$, $p<0.01$.

Note: Data are missing for *N. salinicola* with a microbiome assembled from Friday Harbor incubated at 18°C, as no replicates achieved exponential growth.

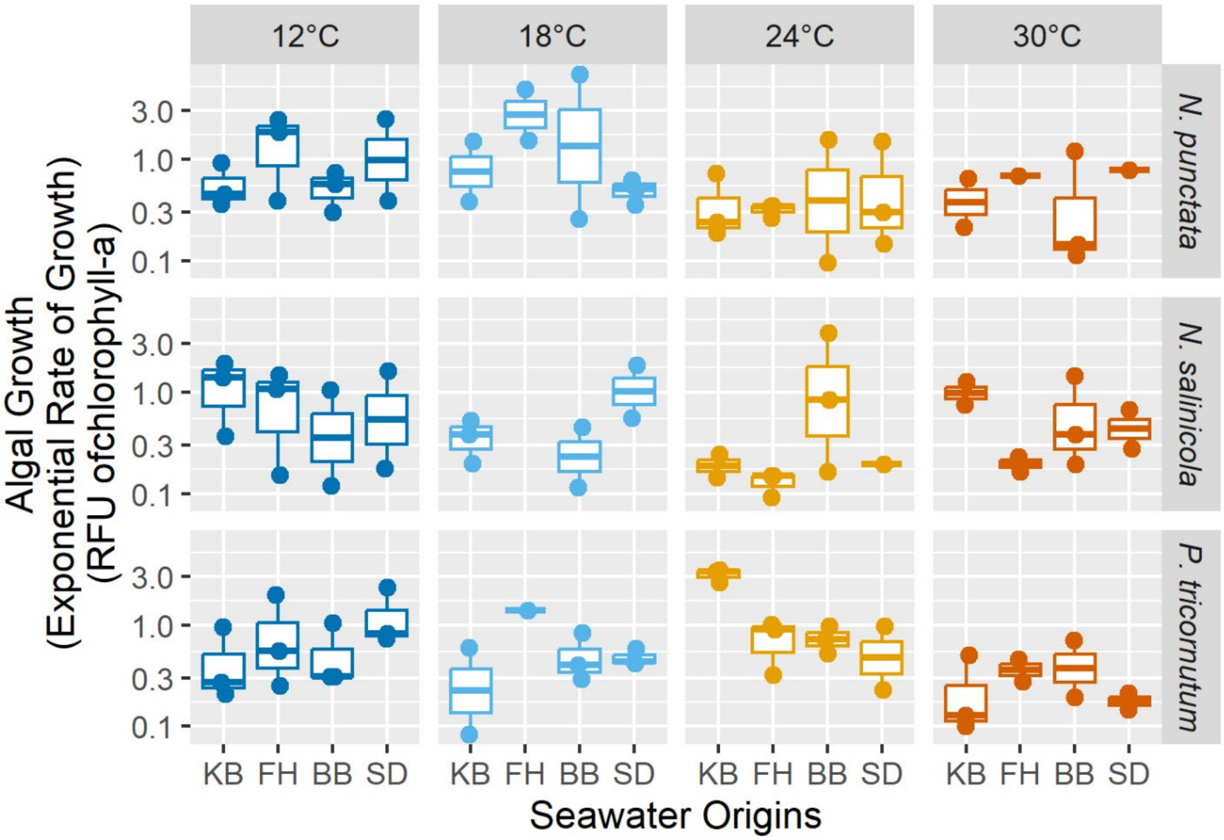

Figure S10. Temperature effects on diatom growth rates across microbiome origins. The incubation temperature significantly influenced the exponential growth rates of certain diatom hosts, while the seawater origin of the assembled microbiome showed fewer significant effects. Three diatom species were inoculated into L1 media after assembling microbiomes from one of four seawater collection sites (KB = Kachemak Bay, Alaska; FH = Friday Harbor, Washington; BB = Bodega Bay, California; SD = San Diego Bay, California) and incubated at one of four temperatures for 29 days. Exponential growth rates were estimated from algal density measured via chlorophyll-a fluorescence, using logistic growth curve modeling ('growthcurver' package in R). Two-way ANOVA was used to evaluate Temperature and Seawater Origin as fixed effects, excluding interaction terms in final models if $p>0.05$. Results are as follows: *Nitzschia punctata*: Temperature- $F_{3,31}=2.13$, $p=0.12$, Seawater Origin- $F_{3,31}=0.76$, $p=0.52$. *Navicula salinicola*: Temperature- $F_{3,29}=0.452$, $p=0.72$, Seawater Origin - $F_{3,29}=0.70$, $p=0.56$. *Phaeodactylum tricornutum*: Temperature- $F_{3,25}=9.29$, $p<0.01$, Seawater Origin- $F_{3,25}=2.79$, $p=0.061$, Temperature x Seawater Origin Interaction $p<0.01$.

Note: Data are missing for *N. salinicola* with a microbiome assembled from Friday Harbor incubated at 18°C, as no replicates achieved exponential growth.

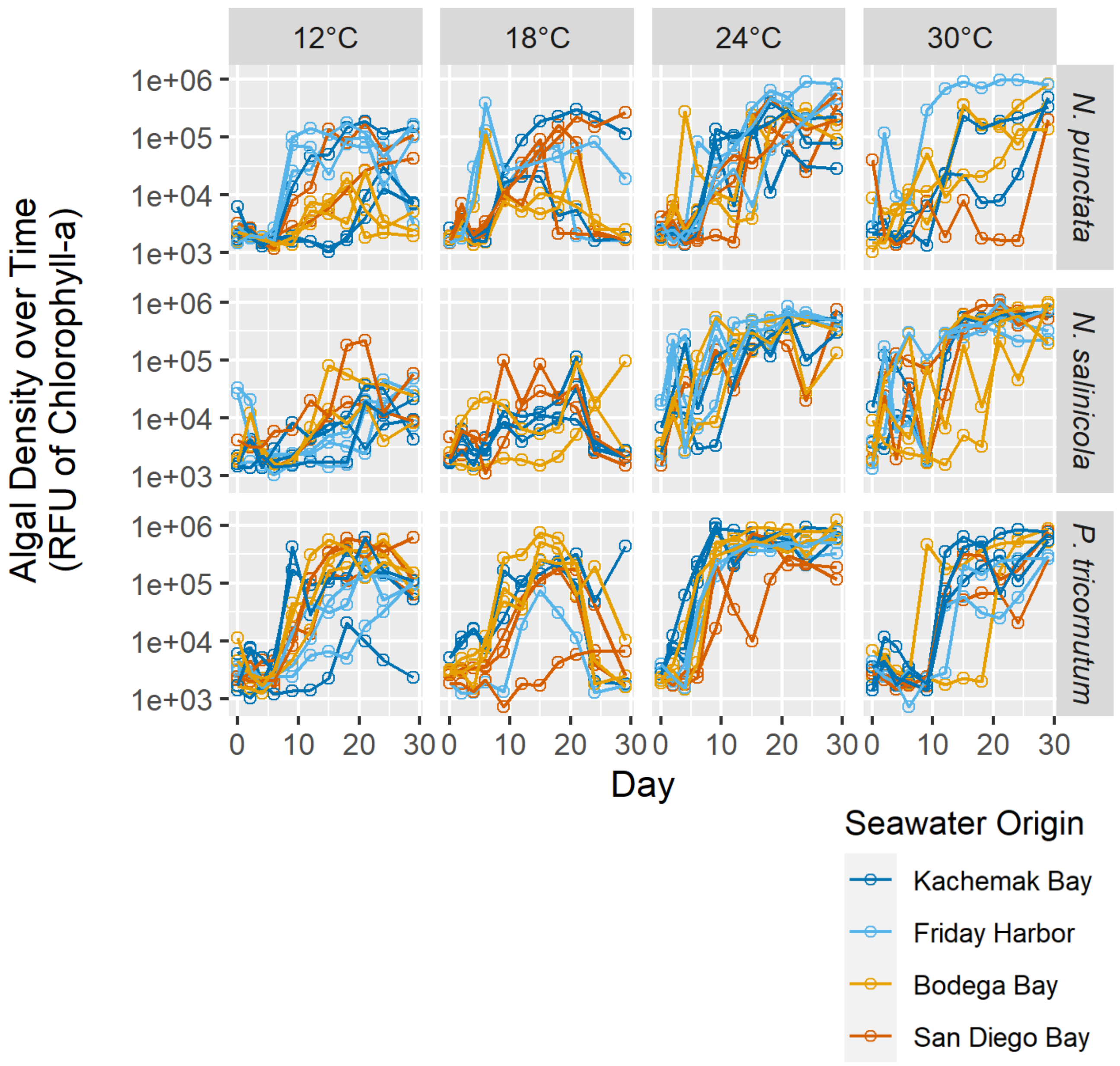

Figure S11. Raw growth trajectories of diatoms across temperatures and locations. Raw measurements of algal growth over time, estimated via chlorophyll-a fluorescence (Relative Fluorescence Units, RFU). Growth curves are shown for three diatom species with microbiomes assembled from seawater collected at four geographic locations: KB = Kachemak Bay, Alaska; FH = Friday Harbor, Washington; BB = Bodega Bay, California; SD = San Diego Bay, California. Cultures were inoculated into L1 media and incubated at one of four temperatures (12ºC, 18ºC, 24ºC, and 30ºC) for 29 days. Each line represents a replicate sample from the corresponding seawater origin and temperature treatment.

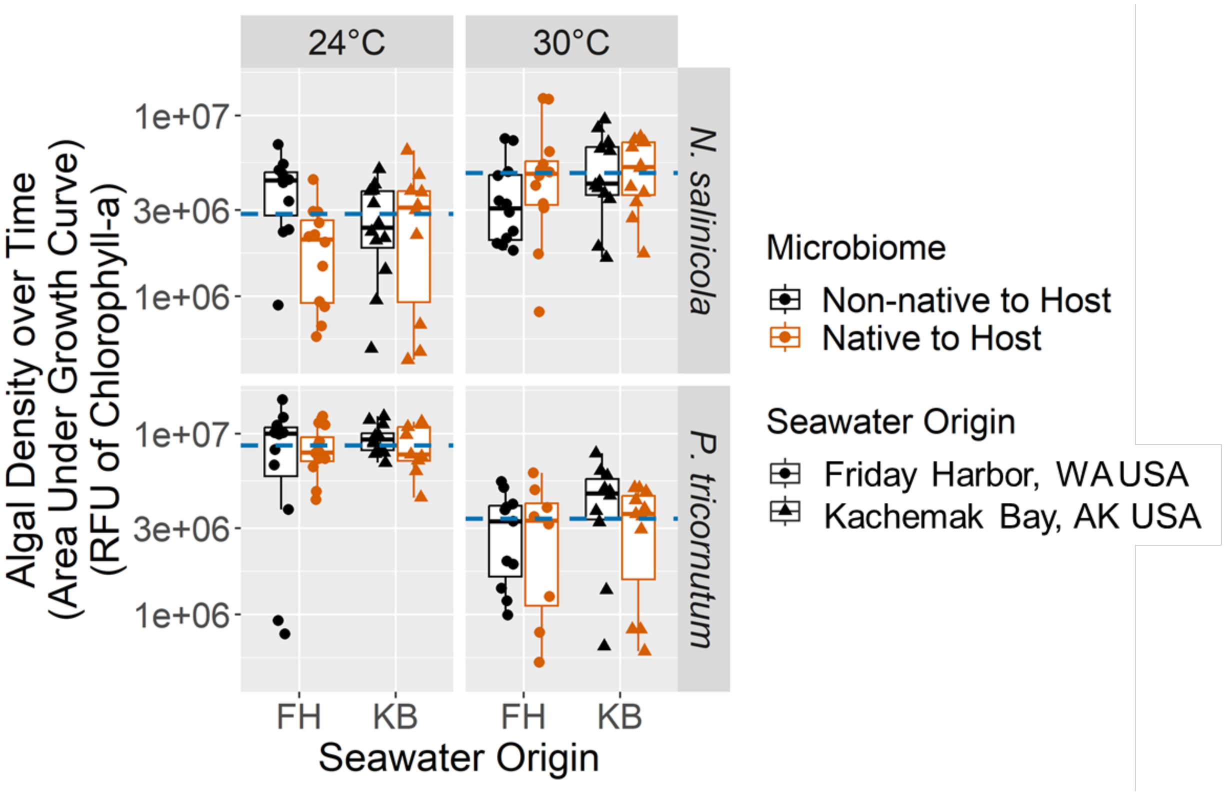

Figure S12. Thermal stress effects on diatom fitness by microbiome type. Host fitness, approximated by population density over time (measured as chlorophyll-a fluorescence), is presented as the total area under fitted logistic growth curves over the 24-day incubation period. Two diatom species (*P. tricornutum* and *N. salinicola*) were inoculated with either their own native microbiome or a microbiome from the other species (non-native). Cultures were incubated at either 24ºC or 30ºC and contained microbiomes assembled from seawater collected at Friday Harbor, Washington (FH), or Kachemak Bay, Alaska (KB). Statistical analyses included an ANOVA with fixed effects of Species-Specific Microbiome (native vs. non-native), Algal Host Species (*P. tricornutum* vs. *N. salinicola*), Seawater Origin (FH vs. KB), and Temperature (24 vs. 30°C). Media type (with vs. without additional vitamin B12) was included as a random effect. Significant effects included Temperature ($F_{1,171}$=123, p<0.01), Algal Host Species ($F_{1,171}$=33.59, p<0.01), and the interaction between Temperature and Algal Host Species (p<0.01). Species-Specific Microbiome ($F_{1,171}$=1.03, p=0.31) and Seawater Origin ($F_{1,171}$=1.72, p=0.19) were not significant. Dashed blue lines represent mean diatom density for each experimental condition.

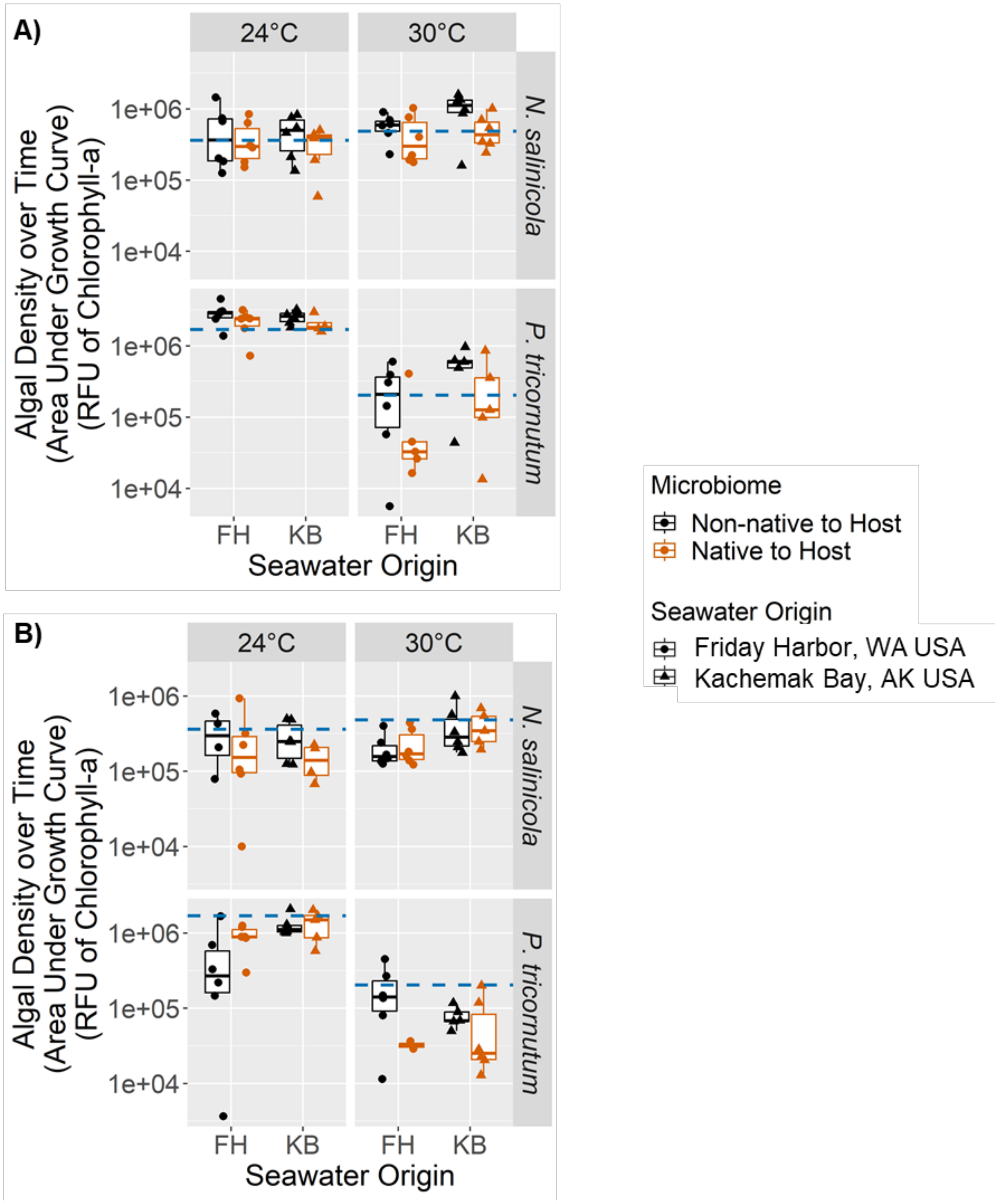

Figure S13. Early-stage growth effects of native vs. non-native microbiomes in diatoms. Early-stage growth dynamics of two diatom species, *N. salinicola* and *P. tricornutum*, as measured via chlorophyll-a fluorescence every 2 – 3 days over the first 12 of a 24-day incubation at two temperatures (24ºC and 30ºC). Axenic diatoms were inoculated with either a microbiome assembled by their own species (“native”), or the other species (“non-native”). Growth conditions included A) L1 media without added vitamin B12 and B) standard L1 media with vitamin B12. Similar trends were observed across media types, and statistical analysis reported in the Fig. 4 legend include media type as a random effects term. Dashed blue lines indicate mean algal density for each panel. Data from the full 24-day growth curve shown in Fig. S12.

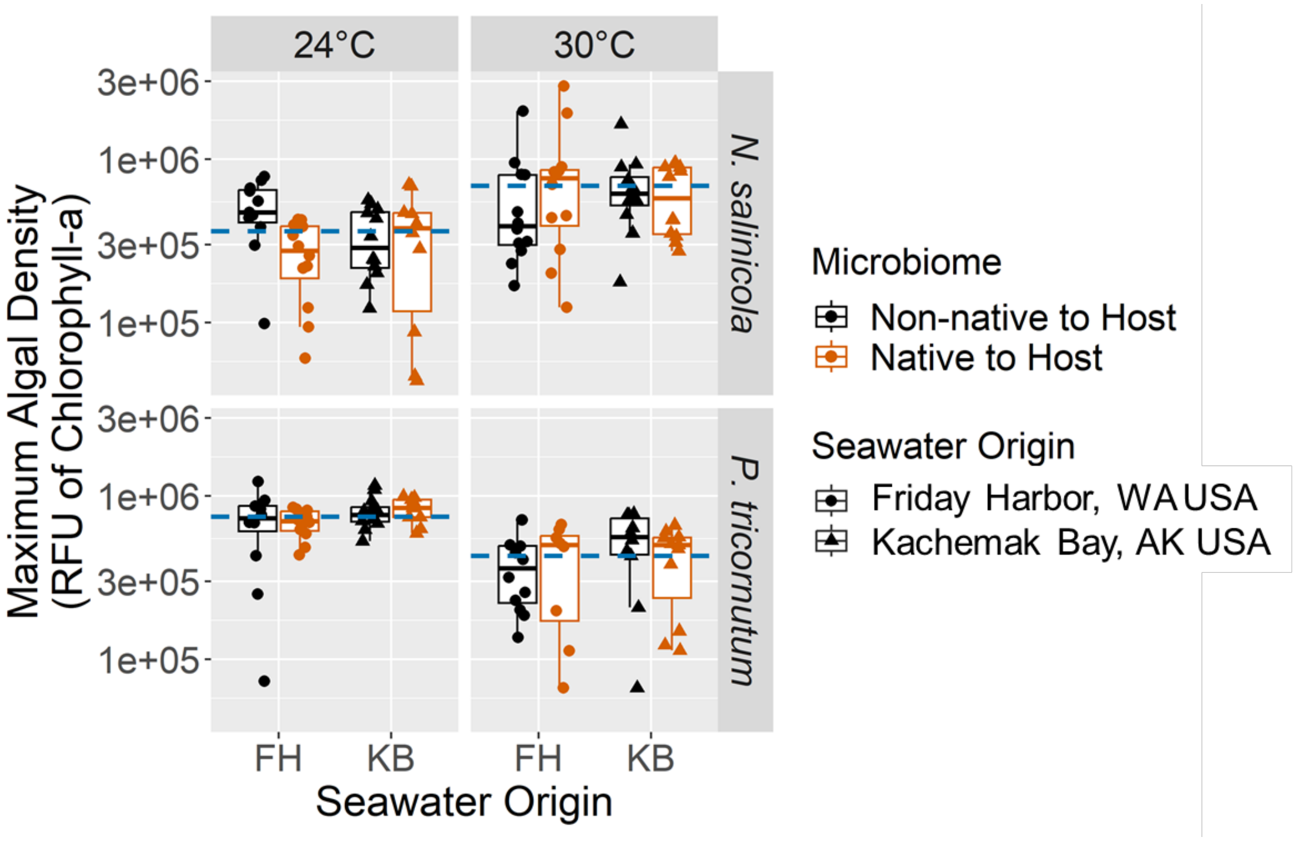

Figure S14. Maximum growth densities of diatoms under native and non-native microbiomes. Host-specific microbiomes exhibited similar effects on the maximum density achieved by each diatom species under thermal stress. Maximum density was identified as the highest RFU value recorded during the 24-day growth period (see complete growth curves in Fig. S16). Axenic diatoms were inoculated with either their own native microbiome, or the microbiome of the other species (i.e., non-native). ANOVA tests were performed to evaluate Species-Specific Microbiomes (i.e., native vs. non-native), Seawater Origin (Friday Harbor, Washington vs. Kachemak Bay, Alaska), and Temperature (24°C vs. 30°C) as fixed effects, using media type as an error term (with or without extra vitamin B12) for each species. *Phaeodactylum tricornutum*: Species-Specific Microbiome: $F_{1,80} = 0.34$, $p = 0.56$; Seawater Origin: $F_{1,80} = 4.41$, $p = 0.039$; Temperature: $F_{1,80} = 61.29$, $p < 0.001$. *Navicula salinicola*: Species-Specific Microbiome: $F_{1,88} = 0.14$, $p = 0.71$; Seawater Origin: $F_{1,88} = 0.089$, $p = 0.77$; Temperature: $F_{1,88} = 18.61$, $p < 0.001$. Dashed blue lines indicate the mean diatom density for each facet.

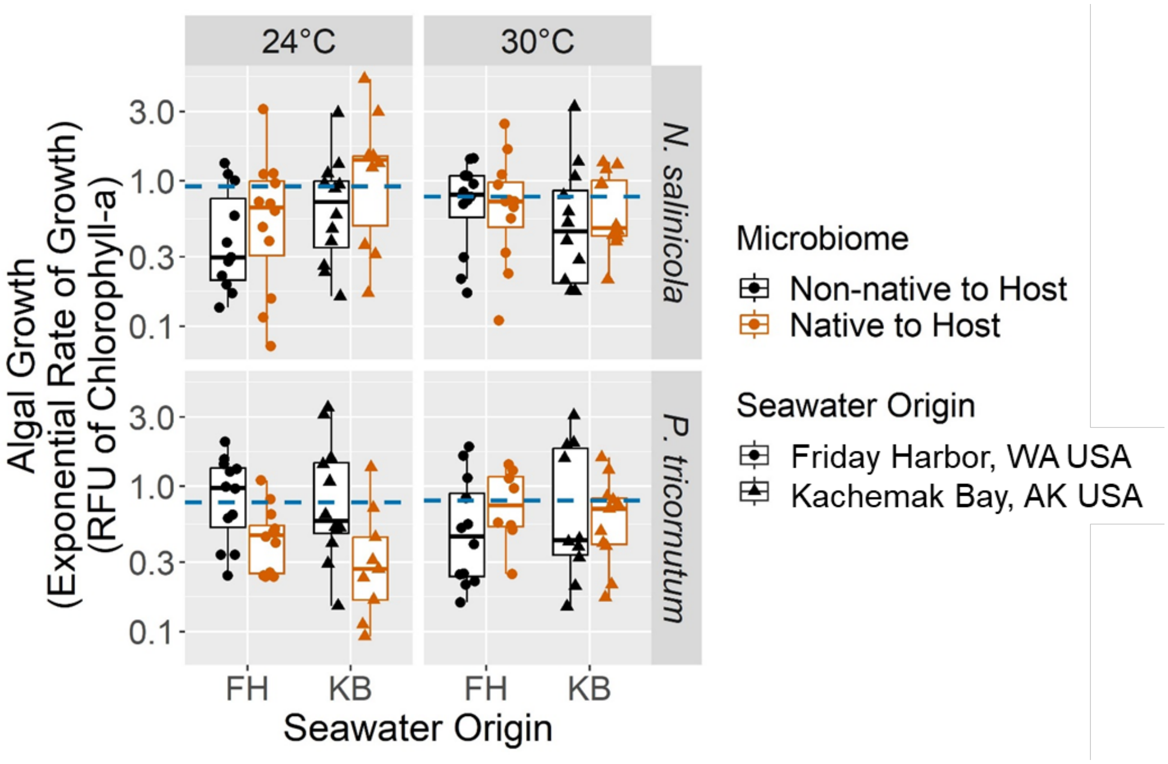

Figure S15. Growth rate effects of native vs. non-native microbiomes in diatoms. Host-specific microbiomes conferred host-specific effects on exponential rate of growth of certain diatom populations under thermal stress. Exponential growth rates were estimated using model fits of diatom density, as measured by chlorophyll-a fluorescence, using the 'growthcurver' package in R. Axenic diatoms were inoculated with either their own native microbiome or the microbiome of the other species (non-native). Analysis of variance (ANOVA) tests assessed the effects of Species-Specific Microbiomes (native vs. non-native), Seawater Origin (Friday Harbor, Washington vs. Kachemak Bay, Alaska), and Temperature (24°C vs. 30°C) with media type (with vs. without extra vitamin B12) included as a random effect. *Phaeodactylum tricornutum*: Species-Specific Microbiome: $F_{1,80}$=5.13, p=0.026; Seawater Origin: $F_{1,80}$=0.14, p=0.71; Temperature: $F_{1,80}$=0.065, p=0.80; Species-Specific Microbiome x Temperature Interaction: $F_{1,80}$=4.86, p=0.030. *Navicula salinicola*: Species-Specific Microbiome: $F_{1,87}$=2.07, p=0.15; Seawater Origin: $F_{1,87}$=1.79, p=0.18; Temperature: $F_{1,87}$=0.95, p=0.33; Seawater Origin x Temperature Interaction: $F_{1,87}$=4.15, p=0.045. Dashed blue lines indicate the mean diatom density for each facet.

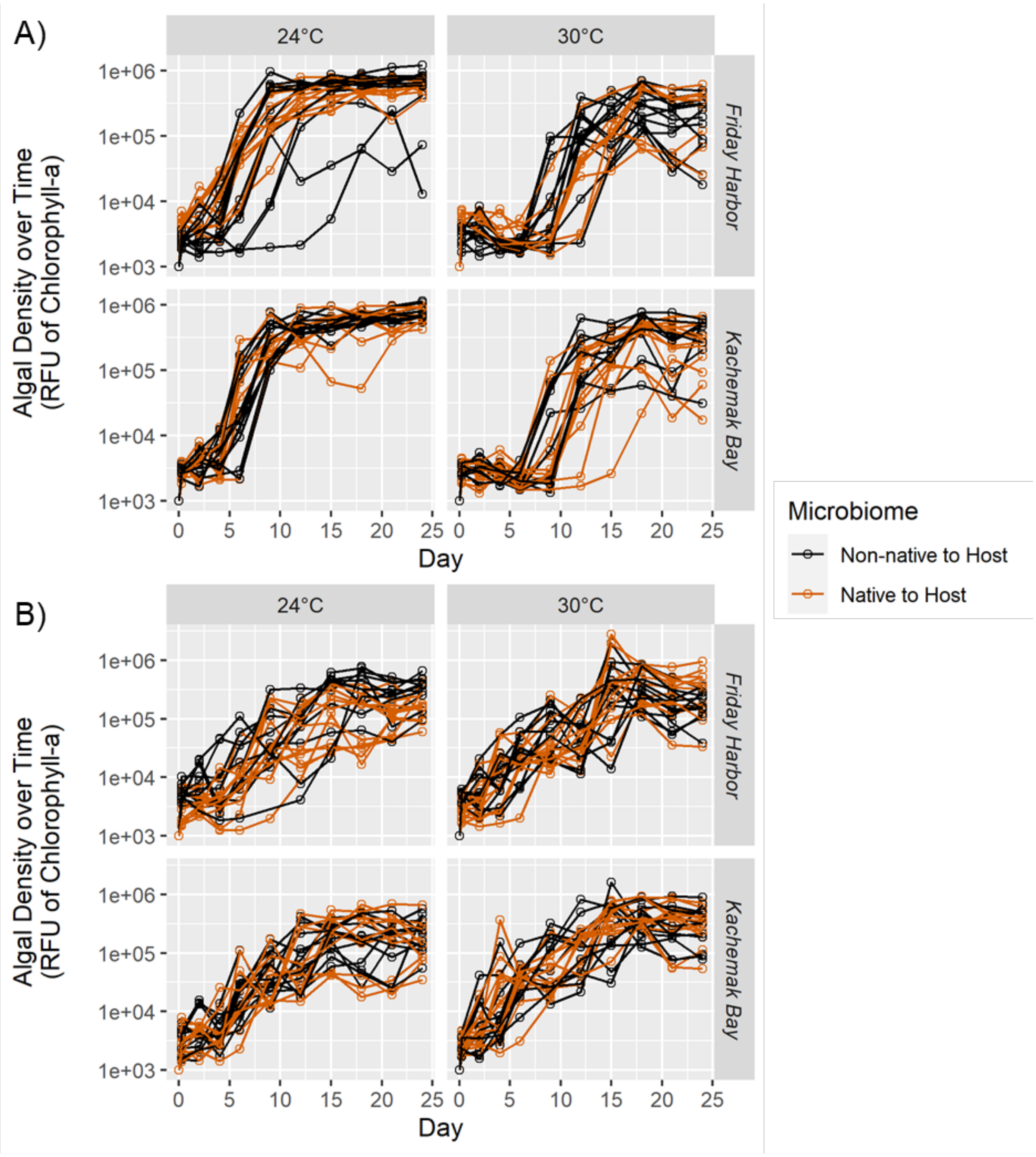

Figure S16. Raw growth trajectories of two diatom species across temperatures. Algal growth trajectories of two diatom species over 24 days, measured via chlorophyll-a fluorescence. Growth measurements represent two diatom species, *P. tricornutum* and *N. salinicola*, inoculated with microbiomes assembled from seawater collected at one of two locations: KB = Kachemak Bay, Alaska; FH = Friday Harbor, Washington. Cultures were inoculated into L1 media at either 24ºC or 30ºC. A) Growth trajectories of *P. tricornutum*. B) Growth trajectories for *N. salinicola*. Data show the effects of native versus non-native microbiomes, highlighting potential host-microbiome interactions under different thermal conditions.

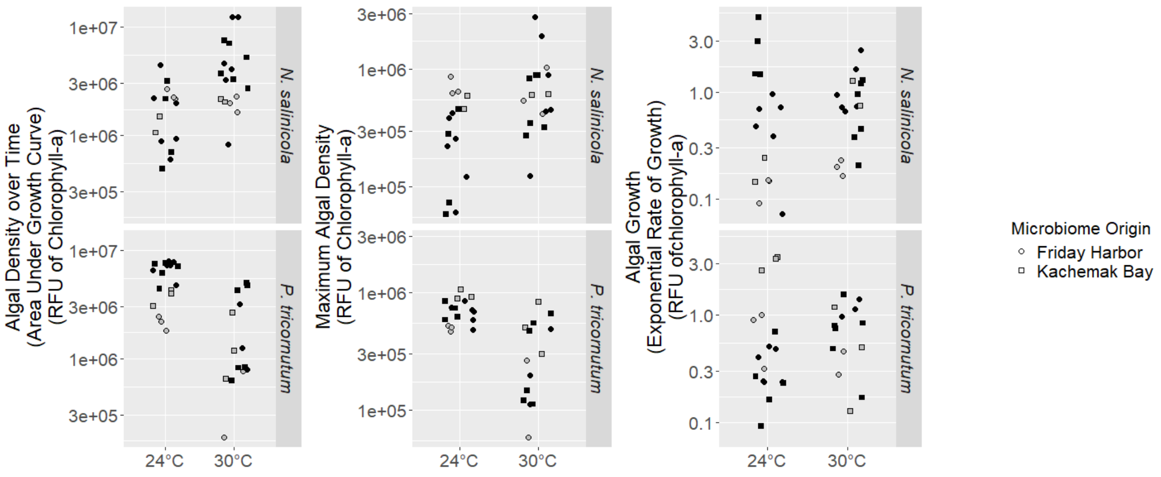

Figure S17. Reproducibility of growth metrics across experiments. Algal density metrics tended to be similar between the Thermal Performance Fitness Assays depicted in gray and the Microbiome Transplant Assays depicted in black, whereas algal growth rates tended to differ between experiments. Specifically, algal density attained over time was similar between experiments for *N. salinicola* ($p > 0.05$), but differed for *P. tricornutum* grown at 24°C (Temperature x Experiment Interaction $p = 0.023$). Maximum algal densities attained were similar between experiments for both species (p-values > 0.05). However, algal growth rates varied between experiments for *N. salinicola* (Experiment $p < 0.01$), and for *P. tricornutum* at 24°C (Temperature x Experiment Interaction $p < 0.01$). To facilitate comparisons between the two studies, only replicates cultured in standard L1 media containing vitamin B12 and those containing their native microbiome were included. Further, Thermal Performance Fitness Assays were reduced to 24-days to enable comparisons with the 24-days Microbiome Transplant Assays.

Varied growth dynamics between studies might be the result of a difference in experimental design— specifically, the length of time permitted for the association between the phytoplankton host and the microbiome prior to starting each experiment. In the Thermal Performance Fitness Assays, bacteria had 18 days to establish associations with their host prior to inoculation for growth curves. In contrast, the Microbiome Transplant Assays necessitated the use of axenic hosts with an introduction of a specific bacterial community. We paired axenic hosts with their microbiome upon inoculation into well-plates on Day-0 of these growth curves. Exponential rates of growth measurements are sensitive to the first few days of growth, when bacterial associations may have still been developing in the Microbiome Transplant Assays. In contrast, we think these associations would have been established by Day-0 of the Thermal Performance Fitness Assays. Maximum Algal Density and Algal Density over Time varied less between experiments, potentially because these metrics are more sensitive to later phases of growth, at which time host-bacterial associations may have become well developed in both experiments. A valuable future direction would be to quantify the effects of this duration of bacterial-host associations on the growth dynamics of the host.
